# Temporal sex specific brain gene expression pattern during early rat embryonic development

**DOI:** 10.3389/fcell.2024.1343800

**Published:** 2024-06-19

**Authors:** Berkay Paylar, Subrata Pramanik, Yared H. Bezabhe, Per-Erik Olsson

**Affiliations:** Biology, The Life Science Center, School of Science and Technology, Örebro University, Örebro, Sweden

**Keywords:** neuronal, sex chromosome, RNA sequencing, sexual dimorphism, differentiation

## Abstract

**Background:** The classical concept of brain sex differentiation suggests that steroid hormones released from the gonads program male and female brains differently. However, several studies indicate that steroid hormones are not the only determinant of brain sex differentiation and that genetic differences could also be involved.

**Methods:** In this study, we have performed RNA sequencing of rat brains at embryonic days 12 (E12), E13, and E14. The aim was to identify differentially expressed genes between male and female rat brains during early development.

**Results:** Analysis of genes expressed with the highest sex differences showed that *Xist* was highly expressed in females having XX genotype with an increasing expression over time. Analysis of genes expressed with the highest male expression identified three early genes, *Sry2, Eif2s3y*, and *Ddx3y*.

**Discussion:** The observed sex-specific expression of genes at early development confirms that the rat brain is sexually dimorphic prior to gonadal action on the brain and identifies *Sry2* and *Eif2s3y* as early genes contributing to male brain development.

## 1 Introduction

Sexual dimorphism, including maternal care, sexual behavior, brain function, structure, and susceptibility to neurological disorders is evident in humans as well as in nonhuman species. Studies of human male and female brains have revealed sex differences in connectome, methylome, and transcriptome profiles (Ingalhalikar et al., 2014; Xu et al., 2014). Despite extensive advancement in neuroscience, the molecular regulation of these sex differences remains unclear.

The classical model of brain sex differentiation that placed gonadal steroid hormones as the main drivers in establishing male and female neural networks was derived from earlier studies (Phoenix et al., 1959; Arnold, 2009). This model states that the chromosomal constitution (XX or XY) determines the gonadal sex and that the hormone secreted by the gonads programs the brain neural network differently (Phoenix et al., 1959; Arnold, 2009).

The initiation of sex differentiation is governed by the sex determining region Y (Sry) master regulator gene located on the Y chromosome, that signals for the activation of the male sex differentiation pathway and the formation of testes (Koopman, 2005). The earliest gonadal expression of Sry is at around E10.5 in mouse, and peaks at E11.5 to initiate testis differentiation (Sim et al., 2008). It has also been observed that Sry is present in the mouse brain at E11 (Mayer et al., 2000). In rats, multiple Sry copies were identified by Turner and co-workers (Turner et al., 2007). They observed that among the different Sry homologues, Sry2 had the highest expression in rat testis and adrenal glands at 15 weeks of age. In a separate study, it was observed that the expression of Sry2 in rat gonads began from E11, as revealed by transcriptional analysis (Prokop et al., 2020). In humans the expression of Sry is observed first at E41 to peak on E44 (Hanley et al., 2000). Studies using the four-core mouse model indicate that Sry may be needed for proper masculinization of the animal, both gonadal and neuronal (Arnold, 2009). A study in chicken also indicate that male and female brains are sexually dimorphic prior to gonadal development (Lee et al., 2009).

Studies on zebra finch has been instrumental in revealing the involvement of genetic elements in neuronal cell sex differentiation. In a study on zebra finch, Gahr and Metzdorf, showed that androgen treatment of female birds did not fully masculinize the song center (Gahr and Metzdorf, 1999). Furthermore, an involvement of genetic factors in brain sex development was evident from a study of a gynandromorphic zebra finch (Arnold, 2003). Despite the whole brain being under the influence of the same gonadal hormones, the zebra finch still developed histologically identifiable song centers in the male side of the brain while the female side of the brain remained feminine (Arnold, 2003). In addition, aromatase inhibitor treatment, that induced testicular tissue in the genetic female zebra finch, failed to masculinize the song system that remained feminine (Wade and Arnold, 1996).

To identify genes involved in brain sexual development Dewing and coworkers performed a study using embryonic mouse heads and microarray analysis (Dewing et al., 2003) They observed sex differences in gene expression at embryonic day 10.5 (E10.5) were observed in the head region. DEAD-box RNA helicase y (Ddx3y; Dby) and eukaryotic translation initiation factor 2 subunit 3y (Eif2s3y) were identified as having the highest male biased gene expression at 10.5 dpc (days post coitum), while X-inactive specific transcript (Xist) showed the highest female bias. While studies on human brain sex differences are limited and generally obtained from mid-gestational developmental stages, they do provide information on brain sex differences (Reinius and Jazin, 2009; Johansson et al., 2016). Analysis of gene expression of sex-chromosome linked genes showed 11 Y-chromosome linked genes that were upregulated in comparison to their X-chromosome homologues in females. Further analysis of two of these genes (Pcdh11y and Nlgn4y) showed that these genes were predominantly expressed in different glial and neuronal cell populations in the CNS. In line with this it has also been shown that the microglia cell displays sex differences with males exhibiting higher microglia count than females throughout the neonatal period (Bordt et al., 2020).

To further elucidate the mechanisms leading to differential transcription in the developing male and female brain, and to identify genes showing sex dependent regulation in the developing rodent brain, we performed a transcriptomic analysis of rat brain during the period of gonadal activation. We selected three embryonic stages to determine sex differences in gene expression in the brain. These were stage E12 prior to gonadal activation (Prokop et al., 2020), E13 at the time of gonadal activation, and at E14 following gonadal activation (Val et al., 2003).

The temporal expression from E12 to E14 revealed two Y-chromosome genes with the highest expression levels at E12, Sry2, and Eif2s3y. As Sry2 showed sex biased gene expression at all three developmental stages, locally expressed Sry cannot be excluded as a regulator of gene expression in the brain prior to testis differentiation. Our results support that there are genetic sex differences in developing brains prior to hormone action and suggest that these differences may be involved in the differential development of male and female brains.

## 2 Materials and methods

### 2.1 Sample processing, genotyping, and RNA sequencing

Brain samples from Sprague Dawley rats (*Rattus norvegicus*) were obtained from Brain Bits (United States). The Spraque Dawley rats represent an outbred strain that was selected to allow for determination of responses related to a normal population of the species. Following sampling the brains were maintained in Hibernate™-E Medium (ThermoFisher Scientific, United States) and were shipped on ice. Directly following receiving the samples they were transferred to −80°C. Three males and three females were used to obtain brain samples from each developmental stage. For genotyping, tissue samples from the body were extracted using a DNA isolation kit (Zymo Research, United States). DNA was quantified using NanoDrop (Denovix, United States) and qPCR was performed for *Xist* and *Sry*, to confirm the sex of the sample and to validate brain specific expression. The primer sequences used for *Sry* and *Xist* are listed in Table 1. The qPCR reaction conditions were as follows: 95°C for 5 min followed by 35 cycles of 95°C for 10 s, 55°C for 15 s, and 72°C for 1 min. The PCR product was run on 1% agarose gel. Following genotyping, three individuals per sex and stage were used for RNA sequencing.

**TABLE 1:** Primers used for qRT-PCR analysis.

Gene name	Gene symbol	Forward (5'→3′)	Reverse (5'→3′)
Sex determining region on the Y chromosome	Sry	gct​gca​cac​cag​tcc​tcc​aag	cag​ggt​cgg​tca​cca​gtg​ata​tca
X-inactive specific transcript	Xist	gga​gtc​gtt​cct​cac​acc​ag	gca​gca​ttc​tgt​cga​gcc​a

The samples were homogenized in Tri Reagent (Sigma) and RNA extraction was performed using Directzol RNA extraction kit (Zymo Research, United States). RNA samples were quantified using NanoDrop (Denovix, United States), and the quality was analyzed using RNA denaturing gel. RNA at a concentration of 50 ng/μL with a OD 260/280 between 1.8 and 2.0, OD 260/230 between 2.0 and 2.2 were sent to GATC Biotech/Eurofins for RNA sequencing. RNA samples with a RNA integrity number equal to or exceeding eight were used for sequencing, Sequencing was performed using Illumina platform to generate 2 × 51 bp reads.

### 2.2 Data analysis

The raw data files were first analyzed for sequence quality using pre-alignment QA/QC. Reads were trimmed from three prime ends based on quality score and the average Phred score for the reads was determined for all replicates. The reads were aligned to the rat genome (Rnor_6.0) using BWA-MEM alignment algorithm followed by quantification to alignment model using Partek genomic software (Partek Inc., St. Louis, United States). BWA was chosen due to its well described alignment accuracy and handling of false negative results which is usually observed by other algorithms with short sequence reads (Wu et al., 2019; Alganmi and Abusamra, 2023). The gene expression counts were normalized using the Counts Per Million reads (CPM) method, followed by 1.0e-4 addition. This adjustment is particularly important for genes located on the Y chromosome, as they do not exhibit any expression in female samples. Lowest gene coverage of 10 was employed to filter out low expressed genes. The resulting normalized counts were used to identify differentially regulated genes using DESeq2 (Anders and Huber, 2010). Analysis of the number of reads of Y chromosome genes was performed by calculating reads per million using Integrated Genomics Viewer version 2.12 (Robinson et al., 2011). Manual inspection of the reads of *Sry* genes was performed to ascertain localization at the correct position and ensured that the low read counts were not merely noise.

Hierarchical clustering and heatmaps were generated for each stage with the average linkage cluster distance metric, in conjunction with the Euclidean point distance metric (https://www.bioinformatics.com.cn/en). Standardized gene expression values (z-scores) were used to visualize each stage. Principal component analysis (PCA) was utilized to visualize relationships between various embryonic stages (Partek Inc., St. Louis, United States). To ensure uniform feature contribution in the construction of the PCA, 18 principal components (normalized counts) underwent a logarithmic transformation with a base of two and a transform offset of 1.

Venn diagrams were individually created using jvenn (https://jvenn.toulouse.inrae.fe/app/index.html) for male and female rats at each developmental stage, illustrating the unique gene sets for each sex. Furthermore, the overlapping genes across different stages were highlighted. Key Y-chromosome gene expression profiles were derived through the utilization of counts per million, allowing for the visualization of gene expression patterns across successive developmental stages.

Bubble plots were created to highlight GO annotations and KEGG pathway analysis for each embryonic stage by employing identified differentially expressed genes (DEGs) using an online data analysis and visualization platform (https://www.bioinformatics.com.cn/en). Terms and pathways were ranked based on their enrichment score and the top 10 enriched terms and pathways are presented. A color gradient was employed to indicate the percentage of sex ratios for the given term. Pathways that were overrepresented in males and females were indicated by red and a green coloring, respectively.

Relationships between proteins encoded by the DEGs at the embryonic stages were visualized using STRING database version 12.0 (Szklarczyk et al., 2019). Networks were individually created for each developmental stage. Interactions were predicted based on a confidence score computed by combining the probabilities from experimental, co-expression, co-occurrence, neighborhood, text mining, and database evidence corrected for the probability of randomly observing an interaction. Interactions with a medium confidence score (0.4) are shown. The thickness of the connecting lines indicates the strength of the interaction. Proteins were assigned to one of three clusters according to their global interaction score. Clusters were created with the KMEANS algorithm. Interactions between clusters are shown by dotted lines. Analysis was performed using False Discovery Rate (FDR <0.1) without limiting to 1.5 times fold change. Gene ontology and pathway enrichment was done in Reactome using Human orthologues (Fabregat et al., 2018) to determine whether the genes were separated into specific categories.

### 2.3 Statistical analysis

Candidate DEGs were initially identified based on a *p*-value threshold of less than 0.05. To control for multiple testing, the FDR step-up method was utilized, with a criterion of an FDR step-up value less than or equal to 0.1. Additionally, a fold change requirement of less than −1.5 or greater than 1.5 was applied to identify DEGs. This approach ensured the selection of genes that are both statistically significant and show substantial changes in expression levels. Significant interactions among gene sets were determined at *p* ≤ 0.05. Statistical analysis was performed using One way ANOVA followed by Tukey’s multiple comparison post-test using the GraphPad Prism eight software (GraphPad software, Boston, United States). The differences were considered significant when the *p*-value was <0.05 (∗*p* < 0.05; ∗∗*p* < 0.01).

## 3 Results

### 3.1 Alignment results

Comprehensive QA/QC results for samples used in this study are shown in Table 2. Key metrics, including coverage, alignment, average coverage depth, average sequence length, average sequence quality, and %GC content, have been summarized. High alignment percentages around 97% indicate robust alignment to the reference genome, ensuring the reliability of the sequencing data. Moreover, the stable average sequence length at approximately 50.97 base pairs emphasized the uniformity of sequenced fragments, that allowed robust downstream data analysis. The average sequence quality scores, ranging from 36.19 to 39.21, reflect the accuracy of base calling, with higher scores indicating lower sequencing errors. Furthermore, the %GC content, consistently ranging from 48.54% to 50.84%, suggested a homogeneous base composition among the samples.

**TABLE 2:** Alignment statistics.

			Average	Average	Average	
Sample	Coverage	Aligned	Depth	Length	Quality	%GC
E12 Male-1	4.88925	97.78	21.7383	50.9759	36.1853	49.0329
E12 Male-2	6.1667	97.94	17.9316	50.9761	36.1916	48.7357
E12 Male-3	5.3285	97.88	17.0218	50.9763	36.1915	49.164
E12 Female-1	6.2791	97.87	21.1414	50.9762	36.1913	48.8289
E12 Female-2	5.72136	97.96	19.2717	50.9765	36.1973	48.8719
E12 Female-3	6.45545	97.92	18.091	50.9764	36.2007	48.6667
E13 Male-1	4.87876	97.66	11.8507	50.9636	38.5991	50.1077
E13 Male-2	4.88408	97.49	11.5121	50.9566	38.3518	50.1377
E13 Male-3	5.27774	97.72	11.9268	50.9632	38.5984	49.4595
E13 Female-1	7.36217	97.76	14.9687	50.9672	38.7602	49.7626
E13 Female-2	5.22015	97.77	11.0533	50.9629	38.591	49.2864
E13 Female-3	5.29042	97.64	10.6814	50.9627	38.5698	49.4276
E14 Male-1	9.24112	97.71	15.558	50.9652	38.6291	50.6518
E14 Male-2	6.60427	97.55	11.7672	50.9691	39.0414	49.8576
E14 Male-3	7.62769	97.65	13.6377	50.9824	39.2096	49.9381
E14 Female-1	9.08841	97.66	17.3476	50.9704	38.9529	49.2044
E14 Female-2	4.68918	97.57	9.4766	50.9624	38.5578	50.8363
E14 Female-3	5.09087	97.67	11.9699	50.9628	38.6121	49.8906

### 3.2 Differentially expressed genes in rat brain

**FIGURE 1:**
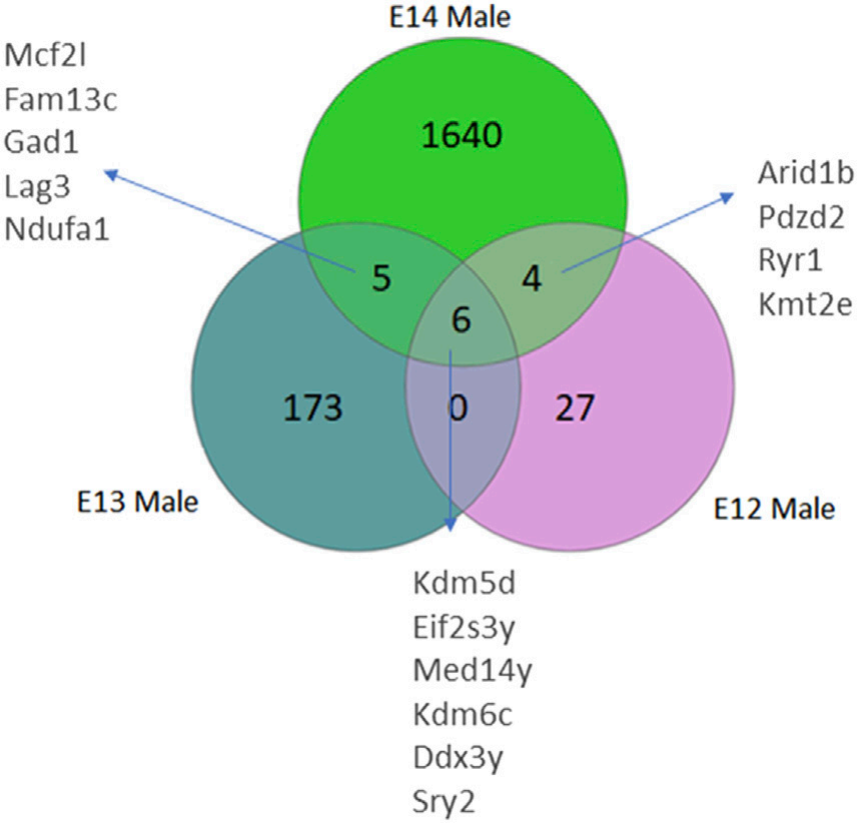
Venn diagrams illustrate the number of differentially expressed genes (DEGs) in male rats, categorized by their developmental stage. DEGs were identified based on an adjusted *p*-value <0.05, following False Discovery Rate (FDR) correction. DEGs common to multiple stages are also annotated.

Analysis of differentially expressed genes in rat brains from E12 to E14 was performed to identify sex differences during early embryogenesis. The number of genes with higher read counts in male brains compared to female brains increased from E12 to E14 (Figure 1; Supplementary Figure S1). At E12, only 37 genes had higher read counts in males, and this number increased to 184 at E13 and 1,655 genes at E14 (Supplementary Table S2). Of these genes, six Y-chromosome genes were differentially expressed at all three developmental stages. In addition, there is an overlap of five DEGs between stages E13 and E14. Furthermore, a set of four genes exhibits differential expression at both E12 and E14. It was interesting to note that there were no overlapping genes between E12 and E13 except for the six Y-chromosome genes.

**FIGURE 2:**
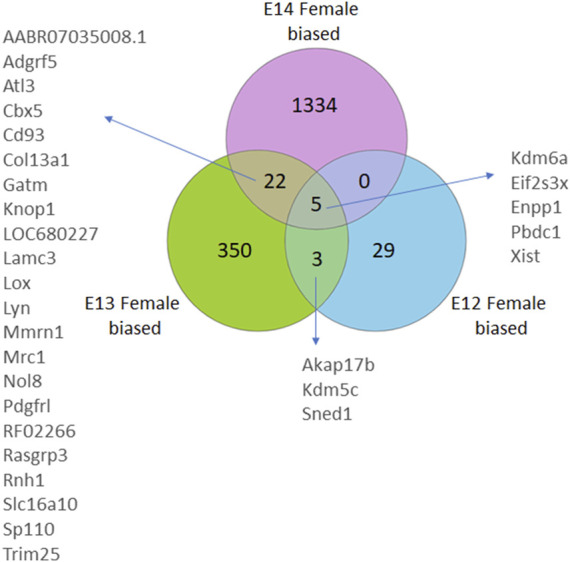
Venn diagrams illustrate the number of differentially expressed genes (DEGs) in female rats, categorized by their developmental stage. DEGs were identified based on an adjusted *p*-value <0.05, following False Discovery Rate (FDR) correction. DEGs common to multiple stages are also annotated.

The gene expression profile in female brains followed a similar pattern with the numbers of female biased genes increasing from E12 to E14 (Figure 2; Supplementary Figure S1). Here, four X-chromosome genes, and one autosomal gene, had significantly higher read counts in female brain at all three developmental stages. Moreover, an overlap of three genes was observed between E12 and E13, and 22 DEGs were common between E13 and E14. Notably, there were no common DEGs between E12 and E14 except for the four X chromosome genes, and one autosomal gene, that overlapped in all three stages.

**FIGURE 3:**
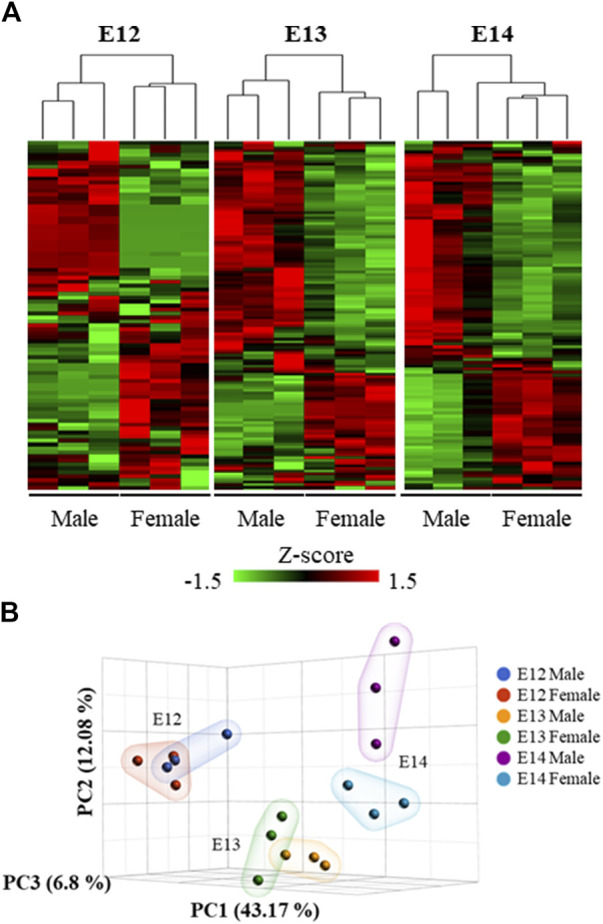
Hierarchical clustering and heat map of the differentially expressed genes. **(A)** The expression patterns at the three studied developmental stages were visualized using standardized gene expression values (z-scores). **(B)** 3D scatter plot illustrates the results of a Principal Component Analysis (PCA) performed on gene expression data from male and female embryos at different developmental stages.

Analysis of the overall gene expression pattern clearly grouped the brains into male and female (Figure 3A). A separation between the sexes at the later stages is apparent from the PCA analysis where the two sexes are clearly separated at E14 (Figure 3B).

**FIGURE 4:**
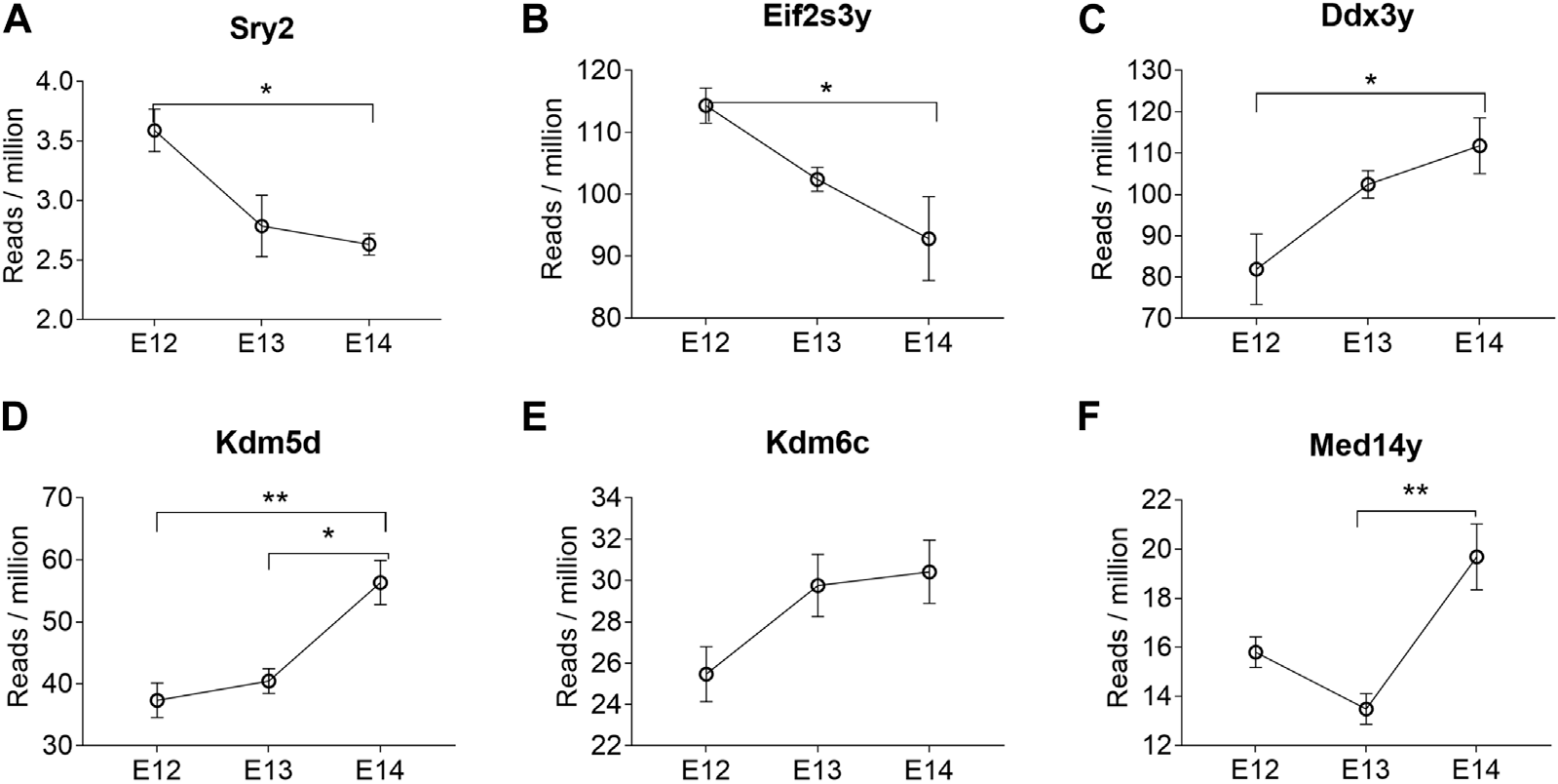
Y chromosome genes differentially expressed at all three developmental stages in male rat brains. The reads per million of DEGs at all three developmental stages are shown. Statistical analysis was performed using One way ANOVA followed by Tukey’s multiple comparison post-test (*p < 0.05 and **p < 0.01) (n = 3, mean ± SEM).

Analysis of abundance and changes in Y chromosome genes was performed by calculating reads per million instead of fold change as these genes are not present in females (Supplementary Figure S2). Manual inspection of the *Sry* reads located at the correct position ensured that the low read counts were not merely noise. The highest expression of the *Sry* genes was observed for *Sry2* (Supplementary Figure S2A). The expression levels of *Sry2* were highest at E12 (Figure 4A). The brain specific expression of *Sry* genes was verified by qPCR, confirming that *Sry* expression was highest at E12 (Supplementary Figure S3). The remaining *Sry* genes showed much lower expression and no stage specific differences (Supplementary Figure S2B-I). It is interesting to note that *Sry2* is located within an *Kdm5d* gene intron in opposite direction of *Kdm5d* (Supplementary Figure S2J). The other Y-chromosome genes that were overlapping between all three stages are shown in Figure 4. Of these, *Eif2s3y* showed a similar expression pattern as *Sry2*, with downregulation from E12 to E14 (Figure 4B). *Ddx3y* (Figure 4C), *Kdm5d* (Figure 4D), and Kdm6c (Figure 4E) showed statistically significant upregulation from E12 to E14. The *Med14y* gene was upregulated from E13 to E14 (Figure 4F), together with the X-chromosome linked *Med14* gene.

**FIGURE 5:**
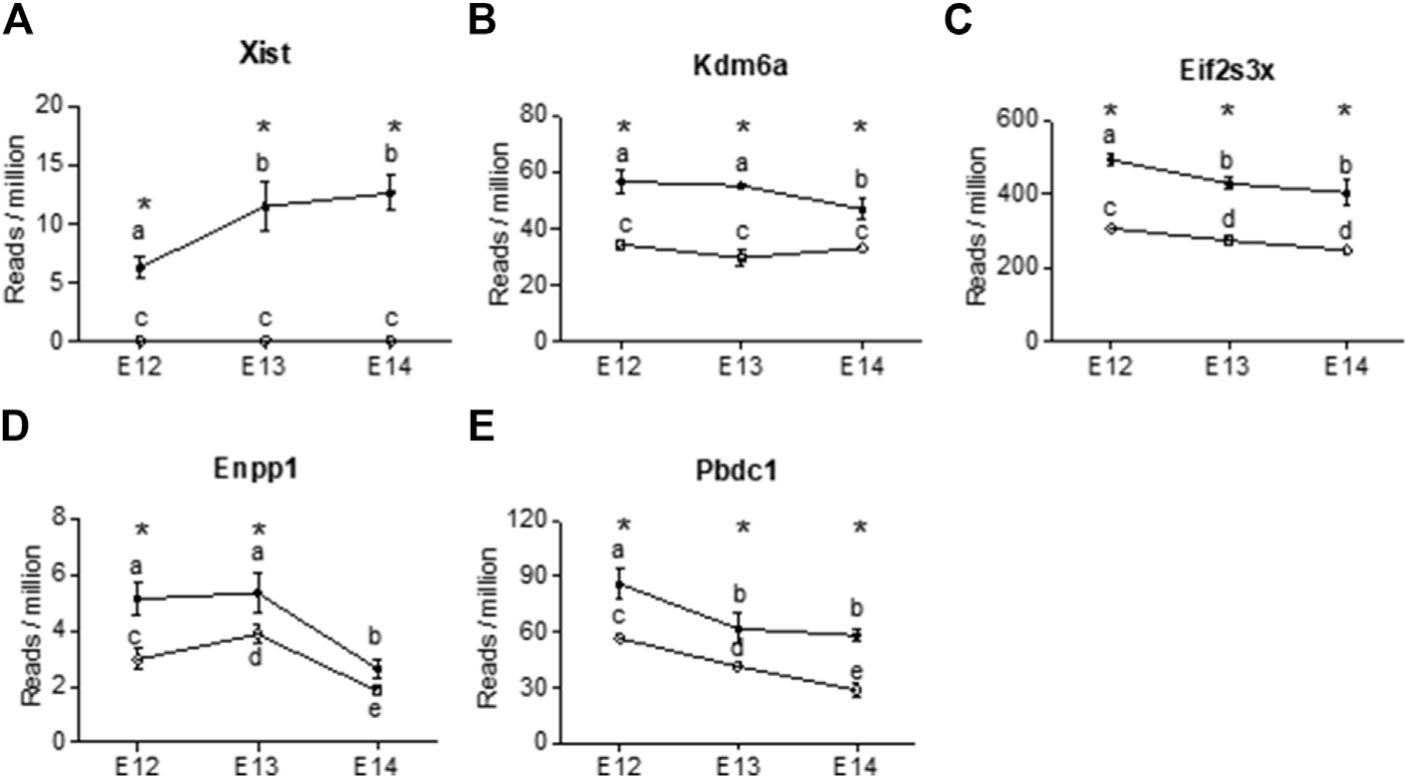
X chromosome genes were differentially expressed at all three developmental stages in female rat brain (closed circles). The reads per million of DEGs at all three developmental stages are shown For male brains (open circles) only three of the genes showed differences in reads per million between developmental stages. **(A)** Xist, **(B)** Kdm6a, **(C)** Eifs2s3x, **(D)** Enpp1, **(E)** Pbdc2. Statistical analysis was performed using One way ANOVA followed by Tukey’s multiple comparison post-test. Different letters were used to denote the level of significance between stages, and asterisk was used to denote statistical differences between sexes at *p* < 0.05 (n = 3, mean ± SEM).

Four X-chromosome linked genes, *Kdm6a*, *Eifs3x*, *Pbdc1* and *Xist* were present at all developmental stages with higher expression in females than in males (Figure 5). Of these, *Xist* was upregulated during embryogenic development in female brain and showed no expression in male brain (Figure 5A). The brain specific expression of *Xist* was verified by qPCR, confirming the upregulation from E12 to E14 (Supplementary Figure S2). *Kdm6a* was only downregulated in female brains at E14 (Figure 5B). *Eif2s3x*, and *Pbdc1* were downregulated in both male and female brains (Figures 5C, E). Also present at all developmental stages was *Enpp1*, coding for an enzyme involved in ATP cleavage, located on chromosome 1. *Enpp1* showed higher expression in female brains at E12 and E13 (Figure 5D). At E14 *Enpp1* was downregulated in both sexes and no sex difference remained. While *Kdm6a* was upregulated at all three developmental stages, the *Kdm5c* gene showed female biased expression at E12 and E13. Both the X-linked *Akap17b* gene and the *Sned1* gene (chromosome 9) showed female biased expression at E12 and E13. There were no common autosomal DEGs between all three developmental stages. However, several autosomal genes associated with proper neuronal functions had higher read counts in male brains at E12 (Supplementary Table S1). These include neuregulin 1 (*Nrg1*, normal development of nervous system), amyloid beta precursor protein (*App*, neural plasticity, and synapse formation), DnaJ Heat Shock Protein Family (*Hsp40*) Member A3 (*Dnaja3*, associated with Alzheimer disease), interleukin one receptor type 1 (*Il1r1*, neuron migration), and neuroligin 1 (*Nlgn1*, synapse function). In addition, ethanolamine kinase 2 (*Etnk2*) which is involved in choline metabolism also had higher read counts in the male rat brain at E12. In female brains, three genes stood out as highly expressed at E12. These were *Somatostatin* (*Sst*), *Cytochrome P450 26b1* (*Cyp26b1*) and (*Cyp2j4*).

### 3.3 Sex and stage specific effects on biological processes

**FIGURE 6:**
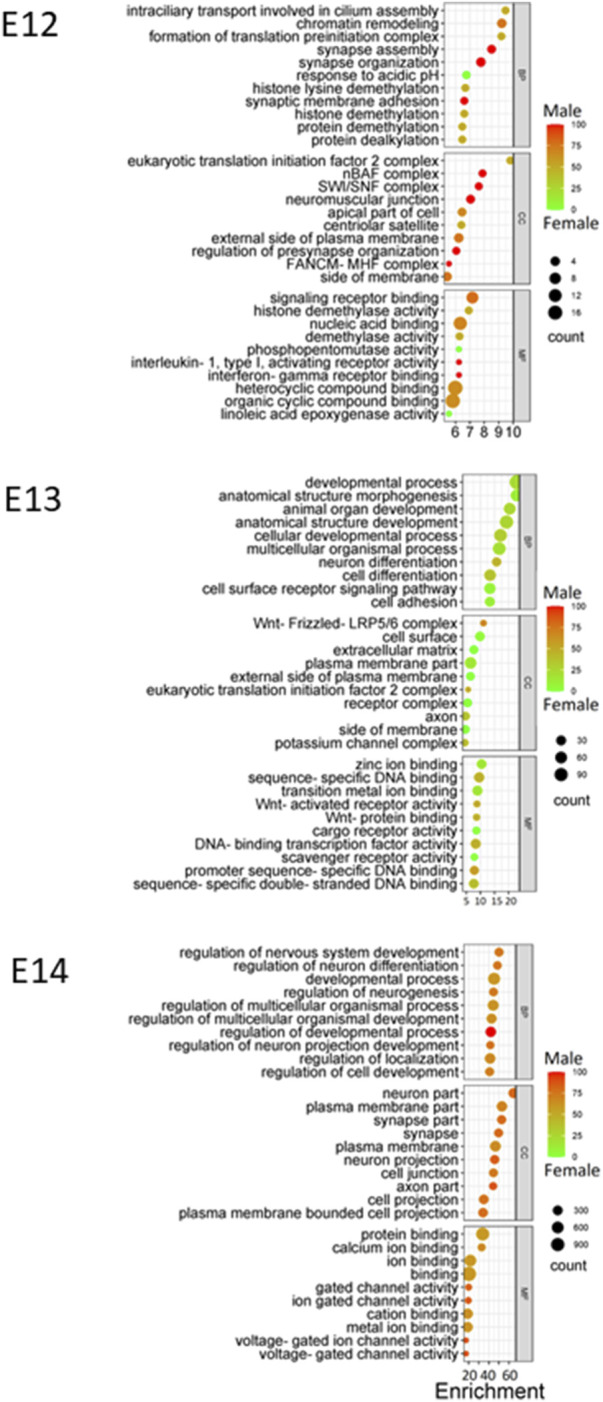
GO enrichment at E12 to E14. Bubble plots were constructed to visually represent Gene Ontology (GO) annotations for each embryonic stage utilizing DEGs identified at each stage, providing a comprehensive overview of the functional categories that are enriched during development. Enriched biological processes (BP), cellular components (CC), and molecular functions (MF) are presented using color gradient to represent the percentage of sex bias for given terms. Red color indicates male dominated enrichment while green color indicates female dominated enrichment.

Gene Ontology (GO) enrichment analysis showed consistent and evolving patterns across the three distinct embryonic stages (Figure 6). Biological processes (BP) crucial to development and regulation, such as developmental process, regulation of nervous system development, and neuron differentiation, exhibited sustained enrichment. However, the interplay of sex-specific influences within these enriched processes emerged when sex ratios were considered for the pathways. At the E12 stage, genes associated with the enriched processes displayed a higher expression in males, emphasizing a potential male gene dominance in these developmental pathways at this point. Interestingly, this dynamic shifted during the E13 stage, with a notable trend towards female gene regulation, suggesting a transitional period in the influence of different sexes. Finally, by the E14 stage, there was a reversion to a male influence, indicating the complexity and variability of sex-specific contributions to development across different stages.

Throughout the embryonic stages, certain cellular components (CC) were consistently enriched. The plasma membrane part, synapse part, and neuron part emerged as recurrently enriched components (Figure 6). Moreover, molecular functions (MF) that encompass binding and receptor activity, including zinc ion binding, sequence-specific DNA binding, and signaling receptor binding, were also consistently enriched across stages. Additionally, the variations in sex percentage involvement across processes, components, and functions provide an intriguing opportunity for further investigation into the interplay between genetics, sex-specific influences, and developmental outcomes.

### 3.4 Brain sex development leads to differentially enriched pathways

The analysis of enriched pathways in the KEGG database showed significant sex differences across the embryonic stages, which is in line with the dynamic nature of embryonic development (Table 3; Supplementary Figure S4). The *p*-values associated with these pathways provided a measure of statistical significance. Some pathways, particularly at later stages, displayed exceptionally low *p*-values, suggesting high statistical significance. In contrast, pathways at E12, such as riboflavin metabolism, exhibited relatively higher *p*-values, indicating less statistically significant associations. The analysis revealed that certain pathways are particularly associated with specific embryonic stages. Notably, embryonic stage E13 was characterized by pathways related to disease and viral infections such as gastric cancer, proteoglycans in cancer, and human papillomavirus infection. In contrast, at embryonic stage E14, neural and cellular processes dominated, with nicotine addiction and glutamatergic synapse showing noteworthy significance. The number of genes associated with these pathways varied significantly between stages and they increased in number as the development of the embryo progressed (Table 3). This was in line with the increasing number of DEGs for consecutive stages. Although the *p*-values and gene counts differed, several pathways, such as extracellular matrix (ECM)-receptor interaction and Focal adhesion, were enriched in both E13 and E14 indicating that these pathways play significant roles in both stages of embryonic development. At embryonic stage E14, a distinct cluster of pathways related to cardiac function and signaling was observed. Pathways such as Hypertrophic cardiomyopathy and Arrhythmogenic right ventricular cardiomyopathy indicated a critical period for the development of the cardiac system during this stage (Table 3).

**TABLE 3:** Enrichment of KEGG pathways among DEGs from E12 to E14.

E12					
Pathway	Enrichment	List	Female biased	Male biased	Male%
Riboflavin metabolism	3.73	7	1	0	0
Inflammatory mediator regulation of TRP channels	3.46	91	1	1	50
Serotonergic synapse	3.26	102	1	1	50
Pantothenate and CoA biosynthesis	2.94	17	1	0	0
E13					
Gastric cancer	11.15	132	8	3	27
Proteoglycans in cancer	9.92	181	10	2	17
Human papillomavirus infection	9.33	292	11	4	27
PI3K-Akt signaling pathway	9.3	293	13	2	13
Signaling pathways regulating pluripotency of stem cells	6.69	126	6	2	25
MicroRNAs in cancer	6.27	135	7	1	13
Hippo signaling pathway	6.18	137	6	2	25
Breast cancer	6.18	137	6	2	25
Cushing’s syndrome	6.14	138	6	2	25
Basal cell carcinoma	5.98	55	3	2	40
Hepatocellular carcinoma	5.28	160	6	2	25
ECM-receptor interaction	4.9	72	5	0	0
Glutamatergic synapse	4.84	104	5	1	17
Pathways in cancer	4.72	468	12	3	20
Focal adhesion	4.5	184	8	0	0
E14					
Nicotine addiction	17.33	20	1	16	94
Glutamatergic synapse	10.25	84	3	23	88
DNA replication	9.39	22	12	0	0
Protein digestion and absorption	9	55	11	8	42
GABAergic synapse	8.29	64	2	18	90
Morphine addiction	7.95	66	2	18	90
Relaxin signaling pathway	7.38	98	11	14	56
Axon guidance	7.31	141	5	27	84
Arrhythmogenic right ventricular cardiomyopathy (ARVC)	6.75	52	6	10	63
Taste transduction	6.48	38	0	13	100
ECM-receptor interaction	6.34	60	14	3	18
Dilated cardiomyopathy (DCM)	6.28	66	6	12	67
Focal adhesion	6.19	159	24	9	27
Hypertrophic cardiomyopathy (HCM)	6.19	61	6	11	65
Synaptic vesicle cycle	6.14	45	1	13	93
Insulin secretion	5.9	63	3	14	82

### 3.5 Predicted stage specific functional protein networks during sex differentiation of the brain

Functional gene network of neural development genes showed significant associations/interactions among differentially expressed genes in the three developmental stages (Supplementary Figure S5). In each stage, three distinct clusters were identified. There were also interactions among protein products within or between neighboring clusters. Interacting proteins contribute to a shared function, an indication of biological interactions. In all developmental stages, Y chromosome genes showed a distinct network and interacted with X-linked genes. At embryonic stage E12, multiple interactions were observed among autosomal proteins involved in signal transduction and neural development and function. In E13, highly expressed genes involved in the development of the reproductive system, produced a distinct network. At embryonic stage E14, a large network of highly expressed genes involved in neural development and gene expression was observed. The reactome analysis furthermore indicates pathway similarities and differences between the three stages (Supplementary Figure S6). At E12 and E13 both developmental biology and signal transduction pathways are enriched, while at E14 developmental and extracellular matrix organization is augmented.

## 4 Discussion

In the present study our aim was to further the understanding of early key events in the process of sex differentiation of the brain. Earlier studies on zebra finch (Agate et al., 2003), mouse (Dewing et al., 2003), and chicken (Lee et al., 2009) suggest involvement of genetic signals in the development of male and female brain neuronal networks. By performing a temporal analysis of gene expression patterns in the early developing brain, around the time of gonadal sex differentiation we were able to determine temporal the regulation of differentially expressed genes. The gene expression pattern gave insight into the increased specialization of the brain, occurring from E12 to E14 and revealed sex biased enriched pathways related to nicotine and morphine addiction.

From experiments using the four-core mouse model it is apparent that Sry is needed for sex differentiation of both gonadal and somatic cells (Arnold, 2009). However, until now it has been unclear if *Sry* is expressed in neuronal cells during sex differentiation of the brain. RNA sequencing of rat embryonic brains revealed the presence of *Sry2* with the highest expression already at the first studied embryonal stage, E12. The expression of *Sry* in rat gonads is detectable at E11 with the expression of *Sry2* followed by the upregulation of other *Sry1*, *Sry3C* and *Sry4a* at E12 (Prokop et al., 2020). At later stages, *Sry4a* appears to be the main form expressed in rat testis (Prokop et al., 2020). Sequence analysis of the different *Sry* isoforms in rat shows that they differ in amino acid sequence with *Sry2* having two point mutations (H4Q and R21H) in the first helix of the HMG box, leading to reduced nuclear translocation (Prokop et al., 2013; Prokop et al., 2016). This has led to questioning the ability of *Sry2* to translocate to the nucleus and activate gene transcription. However, it has been shown that transfection of Chinese hamster ovarian (CHO) cell lines with the *Sry2* transcript results in activation of downstream genes (Milsted et al., 2010; Prokop et al., 2016). In the earlier study *Sry2* was compared to *Sry1* and *Sry3* for its ability to regulate the renin-angiotensin system (Milsted et al., 2010). While *Sry1* and *Sry3* were more potent than *Sry2* it remains that all three isoforms could regulate the renin-angiotensin system. In the later study expression of the *Sry1*, *Sry2* and *Sry3* isoforms in CHO cells resulted in translocation to the nucleus all three isoforms, with *Sry2* showing equal distribution between nucleus and cytosol (Prokop et al., 2016). Thus, while *Sry2* appears to be less efficient at nuclear translocation and activation of downstream gene regulation it still is functional.

In mice testis, *Sry* first appears at 10.5 dpc, peaks at 11.5 dpc, and by 12.5 dpc the expression is undetectable (Larney et al., 2014). *Sry* has previously been reported to be expressed in adult mouse and human male brains (Mayer et al., 1998; Vawter et al., 2004; Dewing et al., 2006; Czech et al., 2012). In male rat gonads, *Sry* expression peaks at E12 (Prokop et al., 2020). However, at this stage the gonad is still underdeveloped (Val et al., 2003). Hence, it can be assumed that steroid secretion is still not initiated from the testis at E13. Thus, it can be concluded that the differential gene expression profiles observed for rat brains at E12 occur prior to hormonal actions on the brain.

In mice brain at E10.5 it was observed that *Eif2s3y* and *Dby* (*Ddx3y*) had the highest fold differential expression in males (Dewing et al., 2003). The remaining genes indicated to be differentially expressed were not located on the Y chromosome (Dewing et al., 2003). In the present study, we identified an increasing number of male biased genes from E12 to E14. There were six Y-chromosome genes showing differential expression at all developmental stages. Besides *Sry2*, we also identified both *Eif2s3y* and *Ddx3y* as early upregulated genes in the male brain. Of these two genes, *Eif2s3y* showed a similar expression profile as *Sry2*, with the highest expression at E12. Overexpression of *Eif2s3y* in mouse neurons has been shown to lead to autism-like behavior in male mice (Zhang et al., 2021). Several autosomal genes (*Nrg1*, *App*, *Dnaja3*, *Il1r1*, *Arid1b*, and *Nlgn1b*) that were upregulated at E12 are also associated with neuronal functions (Mucke et al., 1996; Stefansson et al., 2004; Tabarean et al., 2006; Martinez-Mir et al., 2013; Ka et al., 2016; Zhou et al., 2020). These results show that the early gene expression pattern in male mice is closely connected to neuronal outcomes in adult males.

In the present study we identified four Y chromosome genes, expressed at all three stages, with homologues on the X chromosome that escape X chromosome inactivation. These were *ddx3y*, *eif2s3y*, *kdm5d*, and *kdm6c*. In female brain the X chromosome homologues to three of these genes, *eif2s3x*, *kdm5c*, and *kdm6a* showed female biased expression, suggesting that the Y chromosome homologues could complement the expression of the X chromosome genes. However, it is worth noting that for all four Y chromosome genes it has been established that their functions are overlapping, but not identical, to their X chromosome counterpart (Mayfour et al., 2019; Shen et al., 2022; Dicke et al., 2023; Li et al., 2023; Liu et al., 2023; Rock et al., 2023). Thus, the sex differences in regulation of these genes may lead to sex specific functions. *Ddx3y* showed increasing expression in brain from E12 to E14. *Ddx3y* and *Kdm6c*, are both located in the AZFa region of the Y-chromosome. The AZFa, b, and c regions on the Y-chromosome have been identified to be required for normal spermatogenesis (Vog et al., 1996). In the AZFa region, it has been suggested that *Ddx3y* is the main gene responsible for infertility (Foresta et al., 2000). In male gonads, *Ddx3y* is expressed in spermatogonia before meiosis and *Ddx3x* is expressed in spermatids (Rauschendorf et al., 2014). Deletion of *Ddx3y* disrupts germ cell development and leads to infertility in males (Ramathal et al., 2015; Dicke et al., 2023). The increased expression of *Ddx3y* from E12 to E14 in rat brain indicate that this gene has key functions in male brain development.

*Kdm5d* and *Kdm6c* were both present at all three developmental stages with *Kdm5d* being upregulated from E12 to E14. The functions of both genes have been shown to differ from their X chromosome homologues (Meyfour et al., 2019; Rock et al., 2023). These genes are active demethylases, acting on Lys 27 of histone H3 (Shpargel et al., 2012; Walport et al., 2014). *Kdm6c* shows >88% similarity with *Kdm6a* and has recently been shown to be an active demethylase, demethylating H3K27, but with lower activity than KDM6A (Walport et al., 2014). Kdm6a regulation of H3K27me3 has been indicated to be an important regulator in embryonic stem cells (Welstead et al., 2012). It was also shown that Kdm6c complemented Kdm6a during cellular differentiation. Both genes have been demonstrated to be important for male neurogenesis (Pottmeier et al., 2020).

While few Y-chromosome genes overlapping in males between all three stages, the number of X-chromosome and autosomal genes increased over time in male brains. At E12, *Foxo4* was the only X-chromosome gene upregulated in the male brain. *Foxo4* is a member of the FOXO family that regulates genes involved in metabolism (Liu et al., 2020). In a study on triple knock-out mice, the loss of *Foxo4*, together with *Foxo1* and *Foxo3*, was shown to reduce insulin responses in male, but not female mice (Penniman et al., 2019). In pathway enrichment analysis, Foxo4 and T-box transcription factor 20 (Tbx20) enriched carcinogenesis developmental process in E12, indicating a crucial role of the genes in the early heart development in male. While FOXO4 along with NKX2-5, and MEF2C bind the promoter of the MYOCD (Myocardin) gene, TBX20 and ISL1:LDB1 complex bind the anterior heart field enhancer of the MEF2C gene during cardiogenesis (Takeuchi et al., 2005; Creemers et al., 2006). The role of *Foxo4* in male brain development is of interest for further studies. In female brains *Xist* showed the highest expression with an upregulation from 12 to E13. In addition to *Xist*, *Kdm5c* also showed increased expression at E13. The Y chromosome homologue, kdm5d, showed increased expression first at E14. It is worth noting that *Kdm5c*, but not *Kdm5d*, has been shown to be involved in the activation of *Xist* (Samanta et al., 2022).

Transcriptome analysis revealed that the progression from E12 to E14 resulted in increased specialization of the brain. Following male biased gene expression at E12 there was a shift toward female biased expression at E13 and again a shift back to male biased expression at E14 (Figure 6). A possible rational for this may be that the reprograming of the male brain at E12 requires more time to activate the new downstream genes compared to a continued differentiation of female neurons. Although it has not been explicitly demonstrated, research suggests that cell proliferation rates in the amygdala of rats may be higher in females compared to males (Krebs-Kraft et al., 2010). The GO enrichment analysis reveals dynamic patterns across embryonic stages, highlighting the role of sex in shaping developmental pathways. The consistent enrichment of certain cellular components and molecular functions underscores their importance in embryonic development. The enrichment of biological processes crucial to development and regulation, such as developmental process, regulation of nervous system development, and neuron differentiation, aligns with the understanding that these processes are fundamental to embryogenesis (Zhang et al., 2019).

Analysis of sex biased enriched pathways showed a strong male bias for nicotine and morphine addiction as well as GABAergic and glutamateric synapses at E14. The neuronal signalling is overlapping for these systems, and it has earlier been shown that they exhibit male biased expression in adults (D’Souza and Markou, 2013; Morgan and Ataras, 2022; Medrano et al., 2023). Nicotine exposure can lead to sex-dependent regulation of signalling pathways (Lee et al., 2021). This suggests that the sex biased effects on adults are established already during early development.

The Reactome pathway analysis indicated that the expression of *Rac1* and *Rock1* resulted in enriched semaphorin 4D. Semaphorin 4D axon guidance molecule influence cell migration and axon guidance which are essential for the proper formation and function of the nervous system (Jongbloets et al. 2014). These results show that axon guidance in nervous system development begin at E12. No neuronal development pathways were enriched during E13. We also observed enriched RHOH GTPase cycle, which regulates the proliferation, survival, migration, and engraftment of hematopoietic progenitor cells and specifically T cell development (Li et al., 2002; Yi et al., 2005). Rho GTPase activation is also associated with increased cancer cell invasiveness and associated with the development and progression of various cancers, including breast cancer, lung cancer, and skin cancer (Murray et al., 2014; Butera et al., 2020).

At E14, NCAM signaling for neurite out-growth and the L1CAM interaction axon guidance pathways were significantly overrepresented by DEGs. Three of the 4 DEGs in the NCAM signaling for neurite out-growth, *Cacna1i*, C*acna1d*, and *Cacna1h*, were upregulated at E14. Similarly, four of the 5 DEGs, *Dlg3*, *Dcx*, *Ank3*, and *L1cam*, that enriched the L1CAM interactions were upregulated. The NCAM and L1CAMs are involved in the formation and maintenance of the nervous system. These neural adhesion molecules act as coreceptors for integrins, growth factors and axon guidance/axon pathfinding receptorsduring nervous system development (Schmid and Maness, 2008; Russell and Bashaw, 2018).

In the present study, we have mapped temporal expression patterns in male and female rat brains. The result shows that a limited number of Y-chromosome and X-chromosome genes are involved in all three studied embryonic stages, forming a cluster of interacting genes. In contrast, the expression of autosomal genes was highly stage specific with no gene involved in all three stages. This suggests that once the signaling for sex determination has been activated, there is a progression in functions that will be developed. In addition, the shift from male to female and back to male gene bias from E12 to E14, indicates that the shift from female to male brain requires more time than the direct development of the female brain. The present study reveals that one of the *Sry* genes, *Sry2,* peaked at E12 or earlier, together with *Eif2s3y*. This is followed by the upregulation of *Ddx3y* and *Kdm5d*. The results suggest that *Sry2* is involved in the early sex differentiation cascade for the male brain.

## Data Availability

The datasets presented in this study can be found in online repositories. The names of the repository/repositories and accession number(s) can be found below: https://www.ncbi.nlm.nih.gov/, PRJNA1046089.
