# Supplementary material for: Temporal sex specific brain gene expression pattern during early rat embryonic development
Source: Front Cell Dev Biol. 2024 Jun 19;12:1343800. doi: 10.3389/fcell.2024.1343800 (PMC11219815; doi:10.3389/fcell.2024.1343800)
Supplement: Supplementary file 2 [file Table1.DOCX]

Supplementary Material

**Tables**

**Supplementary Table S1.** Top 10 female and male biased autosomal genes in E12, E13, and E14 developmental stages of Rat brain based on fold change.

**Supplementary Table S2**. Differentially expressed genes at E12, E13 and E14.

**Supplementary Table S3.** Top 12 biomarker autosomal female and male biased genes in rat brain at E12, E13, and E14 developmental stages.

**Figures**

**Supplementary Figure S1**. Venn diagrams displaying DEGs with sex biased expression in male and female rat brains during E12 (**A**), E13 (**B**) and E14 (**C**) stages.

**Supplementary Figure S2.** Copy number and localization of *Sry* genes on the Y chromosome

**Supplementary Figure S3**. qPCR validation of brain specific expression of *Sry* and *Xist* genes.

**Supplementary Figure S4.** Enrichment of KEGG pathways among DEGs from E12 to E14.

**Supplementary Figure S5.** Functional gene network analysis of neural development genes at (**A)** E12, (**B)** E13, and (**C)** E14 with STRING.

**Supplementary Figure S6.** Reactome Voronoi treemaps of the top enriched pathways.

# Supplementary Tables

**Supplementary Table S1.** Top 10 female and male biased autosomal genes in E12, E13, and E14 developmental stages of Rat brain based on fold change.

| **E12 Female Biased Gene Expression** | | | | | | | | |
| --- | --- | --- | --- | --- | --- | --- | --- | --- |
| Feature ID | Chromosome | Total counts | P-value | FDR step up | Ratio | Fold change | LS Mean (Male) | LS Mean (Female) |
| Sst | 11 | 14.86 | <0.01 | <0.01 | 0.17 | -5.92 | 0.65 | 3.88 |
| Cyp26b1 | 4 | 14.47 | <0.01 | 0.05 | 0.33 | -3.05 | 1.12 | 3.43 |
| Cyp2j4 | 5 | 10.55 | <0.01 | 0.09 | 0.38 | -2.61 | 0.94 | 2.47 |
| LOC100361087 | 13 | 19.71 | <0.01 | 0.03 | 0.49 | -2.04 | 2.12 | 4.33 |
| Pcm1 | 16 | 13.74 | <0.01 | 0.02 | 0.55 | -1.83 | 1.60 | 2.93 |
| Sncaip | 18 | 71.18 | <0.01 | 0.02 | 0.57 | -1.74 | 8.58 | 14.93 |
| Enpp1 | 1 | 24.29 | <0.01 | <0.01 | 0.58 | -1.73 | 2.96 | 5.10 |
| Apbb2 | 14 | 13.95 | <0.01 | 0.03 | 0.63 | -1.60 | 1.78 | 2.84 |
| Pgm2 | 14 | 54.24 | <0.01 | 0.03 | 0.65 | -1.53 | 7.10 | 10.90 |
| Sned1 | 9 | 10.02 | <0.01 | 0.07 | 0.66 | -1.52 | 1.32 | 2.00 |
| **E12 Male Biased Gene Expression** | | | | | | | | |
| Nrg1 | 16 | 10.43 | <0.01 | <0.01 | 3.71 | 3.71 | 2.56 | 0.69 |
| App | 11 | 18.45 | <0.01 | <0.01 | 3.05 | 3.05 | 4.51 | 1.48 |
| Ssx2ip | 2 | 10.34 | <0.01 | <0.01 | 2.82 | 2.82 | 2.52 | 0.89 |
| LOC102548286 | 13 | 13.98 | <0.01 | <0.01 | 1.91 | 1.91 | 3.02 | 1.58 |
| Smarce1 | 10 | 134.46 | <0.01 | <0.01 | 1.75 | 1.75 | 28.39 | 16.20 |
| Tox4 | 15 | 23.47 | <0.01 | <0.01 | 1.71 | 1.71 | 4.89 | 2.86 |
| Etnk2 | 13 | 28.40 | <0.01 | <0.01 | 1.71 | 1.71 | 5.94 | 3.48 |
| Il1r1 | 9 | 12.87 | <0.01 | <0.01 | 1.70 | 1.70 | 2.68 | 1.57 |
| Nlgn1 | 2 | 39.59 | <0.01 | <0.01 | 1.67 | 1.67 | 8.23 | 4.91 |
| Dnaja3 | 10 | 122.98 | <0.01 | <0.01 | 1.65 | 1.65 | 25.49 | 15.42 |

| **E13 Female Biased Gene Expression** | | | | | | | | | |
| --- | --- | --- | --- | --- | --- | --- | --- | --- | --- |
| Feature ID | Chromosome | Total counts | P-value | FDR step up | Ratio | Fold change | LS Mean (Male) | LS Mean (Female) |  |
| Supt20h | 2 | 20.62 | <0.01 | 0.04 | 0.11 | -9.33 | 0.64 | 5.93 |  |
| Cald1 | 4 | 36.85 | <0.01 | 0.03 | 0.18 | -5.64 | 1.72 | 9.70 |  |
| Prph | 7 | 30.05 | <0.01 | <0.01 | 0.21 | -4.68 | 1.74 | 8.13 |  |
| Pax7 | 5 | 22.22 | <0.01 | <0.01 | 0.21 | -4.68 | 1.28 | 6.00 |  |
| Corin | 14 | 20.11 | <0.01 | <0.01 | 0.23 | -4.38 | 1.22 | 5.34 |  |
| Rnh1 | 1 | 15.89 | <0.01 | <0.01 | 0.23 | -4.27 | 0.99 | 4.23 |  |
| Mis18bp1 | 6 | 16.30 | <0.01 | 0.01 | 0.25 | -4.08 | 1.01 | 4.11 |  |
| Tfap2b | 9 | 421.83 | <0.01 | 0.05 | 0.25 | -3.99 | 26.58 | 106.06 |  |
| Crabp1 | 8 | 782.46 | <0.01 | 0.05 | 0.28 | -3.63 | 50.10 | 181.72 |  |
| Plxna4 | 4 | 16.21 | <0.01 | 0.08 | 0.28 | -3.59 | 1.12 | 4.03 |  |
| **E13 Male Biased Gene Expression** | | | | | | | | | |
| Nfyc | 5 | 10.55 | <0.01 | 0.07 | 3.10 | 3.10 | 2.52 | 0.81 |  |
| Mx2 | 11 | 10.61 | <0.01 | 0.02 | 3.09 | 3.09 | 2.57 | 0.83 |  |
| Smg6 | 10 | 16.00 | <0.01 | 0.02 | 2.94 | 2.94 | 3.89 | 1.32 |  |
| Thumpd2 | 6 | 14.60 | <0.01 | <0.01 | 2.52 | 2.52 | 3.43 | 1.36 |  |
| RF00017 | 6 | 16.12 | <0.01 | <0.01 | 2.50 | 2.50 | 3.83 | 1.53 |  |
| Neurod6 | 4 | 45.00 | <0.01 | <0.01 | 2.39 | 2.39 | 10.42 | 4.36 |  |
| Fam13c | 20 | 12.04 | <0.01 | 0.01 | 2.38 | 2.38 | 2.77 | 1.16 |  |
| Ppp2r2b | 18 | 24.84 | <0.01 | <0.01 | 2.11 | 2.11 | 5.60 | 2.65 |  |
| Neurod2 | 10 | 43.39 | <0.01 | <0.01 | 2.05 | 2.05 | 9.62 | 4.68 |  |
| Gsx2 | 14 | 48.87 | <0.01 | <0.01 | 2.03 | 2.03 | 10.77 | 5.31 |  |

| **E14 Female Biased Gene Expression** | | | | | | | | | |
| --- | --- | --- | --- | --- | --- | --- | --- | --- | --- |
| Feature ID | Chromosome | Total counts | P-value | FDR step up | Ratio | Fold change | LS Mean (Male) | LS Mean  (Female) |  |
| Ank2 | 2 | 31.34 | <0.01 | <0.01 | 0.00 | -610.57 | 0.02 | 9.92 |  |
| Stat6 | 7 | 18.48 | <0.01 | 0.03 | 0.04 | -23.88 | 0.22 | 5.32 |  |
| Fmo1 | 13 | 20.37 | 0.01 | 0.07 | 0.04 | -22.64 | 0.25 | 5.59 |  |
| Tpm1 | 8 | 11.64 | <0.01 | 0.04 | 0.08 | -12.11 | 0.26 | 3.11 |  |
| Wdr63 | 2 | 11.21 | 0.02 | 0.09 | 0.08 | -12.03 | 0.25 | 2.96 |  |
| Lyve1 | 1 | 60.08 | 0.01 | 0.08 | 0.09 | -11.00 | 1.47 | 16.12 |  |
| Clec18a | 19 | 10.06 | <0.01 | <0.01 | 0.12 | -8.47 | 0.33 | 2.77 |  |
| AABR07019383.1 | 15 | 72.82 | 0.01 | 0.08 | 0.13 | -7.77 | 2.46 | 19.09 |  |
| Lamc3 | 3 | 43.15 | 0.02 | 0.10 | 0.13 | -7.71 | 1.46 | 11.24 |  |
| Lum | 7 | 252.33 | 0.01 | 0.08 | 0.14 | -7.07 | 9.50 | 67.14 |  |
| **E14 Male Biased Gene Expression** | | | | | | | | | |
| Hoxb2 | 10 | 69.26 | <0.01 | <0.01 | 38645.78 | 38645.78 | 3.86 | 0.00 |  |
| Tlx3 | 10 | 19.58 | <0.01 | <0.01 | 3162.42 | 3162.42 | 1.83 | 0.00 |  |
| Foxa2 | 3 | 28.65 | 0.01 | 0.08 | 3060.74 | 3060.74 | 8.24 | 0.00 |  |
| Lbx1 | 1 | 37.20 | 0.01 | 0.06 | 2317.31 | 2317.31 | 1.93 | 0.00 |  |
| Ccnt2 | 13 | 11.80 | 0.01 | 0.05 | 372.55 | 372.55 | 1.36 | 0.00 |  |
| Pou4f2 | 19 | 44.10 | <0.01 | 0.01 | 81.86 | 81.86 | 5.90 | 0.07 |  |
| C1ql4 | 7 | 41.08 | <0.01 | <0.01 | 51.94 | 51.94 | 10.16 | 0.20 |  |
| En2 | 4 | 189.86 | <0.01 | 0.04 | 47.29 | 47.29 | 23.05 | 0.49 |  |
| Gata3 | 17 | 95.30 | <0.01 | <0.01 | 32.87 | 32.87 | 25.41 | 0.77 |  |
| Skor2 | 18 | 25.50 | <0.01 | 0.01 | 20.19 | 20.19 | 4.76 | 0.24 |  |

**Supplementary Table S3.** Top 12 biomarker autosomal female and male biased genes in rat brain at E12, E13, and E14 developmental stages.

| E12 | | | | | | | |
| --- | --- | --- | --- | --- | --- | --- | --- |
| ID | Total counts | p-value | FDR | Ratio | Fold Change | LS Male | LS Female |
| Ugt1a7c | 7.71 | <0.01 | <0.01 | 0.34 | -2.91 | 0.65 | 1.90 |
| Cklf | 7.66 | <0.01 | <0.01 | 0.66 | -1.51 | 1.01 | 1.53 |
| Pld1 | 7.79 | <0.01 | <0.01 | 0.39 | -2.58 | 0.72 | 1.86 |
| Tmprss5 | 7.18 | <0.01 | <0.01 | 0.47 | -2.11 | 0.77 | 1.62 |
| Enpp1 | 24.29 | <0.01 | <0.01 | 0.58 | -1.73 | 2.96 | 5.10 |
| Sst | 14.86 | <0.01 | <0.01 | 0.17 | -5.92 | 0.65 | 3.88 |
| Smarce1 | 134.46 | <0.01 | <0.01 | 1.75 | 1.75 | 28.39 | 16.20 |
| Nlgn1 | 39.59 | <0.01 | <0.01 | 1.67 | 1.67 | 8.23 | 4.91 |
| Dnaja3 | 122.98 | <0.01 | <0.01 | 1.65 | 1.65 | 25.49 | 15.42 |
| Ssx2ip | 10.34 | <0.01 | <0.01 | 2.82 | 2.82 | 2.52 | 0.89 |
| Etnk2 | 28.40 | <0.01 | <0.01 | 1.71 | 1.71 | 5.94 | 3.48 |
| Macrod1 | 9.38 | <0.01 | <0.01 | 1.83 | 1.83 | 2.02 | 1.10 |
| E13 | | | | | | | |
| ID | Total counts | p-value | FDR | Ratio | Fold Change | LS Male | LS Female |
| Enpp2 | 3.83 | <0.01 | <0.01 | 0.47 | -2.11 | 1.48 | 3.12 |
| Tifab | 10.64 | <0.01 | <0.01 | 0.62 | -1.60 | 1.36 | 2.18 |
| Itga1 | 0.84 | <0.01 | <0.01 | 0.65 | -1.53 | 1.43 | 2.18 |
| Pdzrn3 | 213.45 | <0.01 | <0.01 | 0.66 | -1.50 | 28.41 | 42.73 |
| Kcna3 | 9.14 | <0.01 | <0.01 | 0.53 | -1.89 | 1.05 | 1.99 |
| Flrt2 | 114.26 | <0.01 | <0.01 | 0.57 | -1.75 | 13.84 | 24.17 |
| Col6a4 | 9.71 | <0.01 | <0.01 | 1.97 | 1.97 | 2.14 | 1.09 |
| Tbr1 | 356.49 | <0.01 | <0.01 | 1.83 | 1.83 | 76.69 | 41.82 |
| Igsf11 | 249.46 | <0.01 | <0.01 | 1.56 | 1.56 | 50.70 | 32.42 |
| Shisa2 | 94.13 | <0.01 | <0.01 | 1.80 | 1.80 | 20.14 | 11.18 |
| Id4 | 1456.00 | <0.01 | <0.01 | 1.55 | 1.55 | 294.66 | 190.03 |
| Eomes | 316.21 | <0.01 | <0.01 | 1.79 | 1.79 | 67.54 | 37.77 |
| E14 | | | | | | | |
| ID | Total counts | p-value | FDR | Ratio | Fold Change | LS Male | LS Female |
| AABR07071440.1 | 34.49 | <0.01 | <0.01 | 0.59 | -1.69 | 4.27 | 7.21 |
| Fam109b | 19.41 | <0.01 | <0.01 | 0.43 | -2.32 | 1.95 | 4.51 |
| S100a11 | 42.17 | <0.01 | <0.01 | 0.55 | -1.80 | 5.01 | 9.03 |
| Itgal | 18.19 | <0.01 | <0.01 | 0.61 | -1.63 | 2.30 | 3.76 |
| Wnk4 | 18.21 | <0.01 | <0.01 | 0.36 | -2.79 | 1.59 | 4.45 |
| Pard3 | 152.74 | <0.01 | <0.01 | 0.55 | -1.80 | 18.14 | 32.69 |
| Tfap2d | 22.71 | <0.01 | <0.01 | 9.18 | 9.18 | 6.40 | 0.70 |
| Mfap3l | 8.53 | <0.01 | <0.01 | 1.63 | 1.63 | 1.76 | 1.08 |
| Lss | 83.43 | <0.01 | <0.01 | 1.59 | 1.59 | 17.03 | 10.74 |
| Faah | 7.39 | <0.01 | <0.01 | 2.56 | 2.56 | 1.76 | 0.69 |
| Pianp | 40.93 | <0.01 | <0.01 | 1.90 | 1.90 | 8.92 | 4.70 |
| Fam8a1 | 187.58 | <0.01 | <0.01 | 1.71 | 1.71 | 39.45 | 23.02 |

# Supplementary Figures


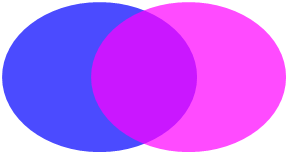


37

16260

37

Male
biased

Female biased


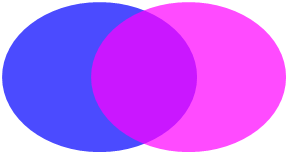


184

16787

380

Male
biased

Female biased


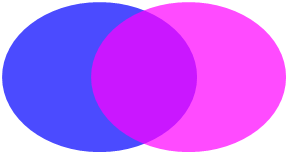


1655

14714

1362

Male
biased

Female biased

**A**

**B**

**C**

**Supplementary Figure S1**. Venn diagrams displaying DEGs with sex biased expression in male and female rat brains during E12 (**A**), E13 (**B**) and E14 (**C**) stages.


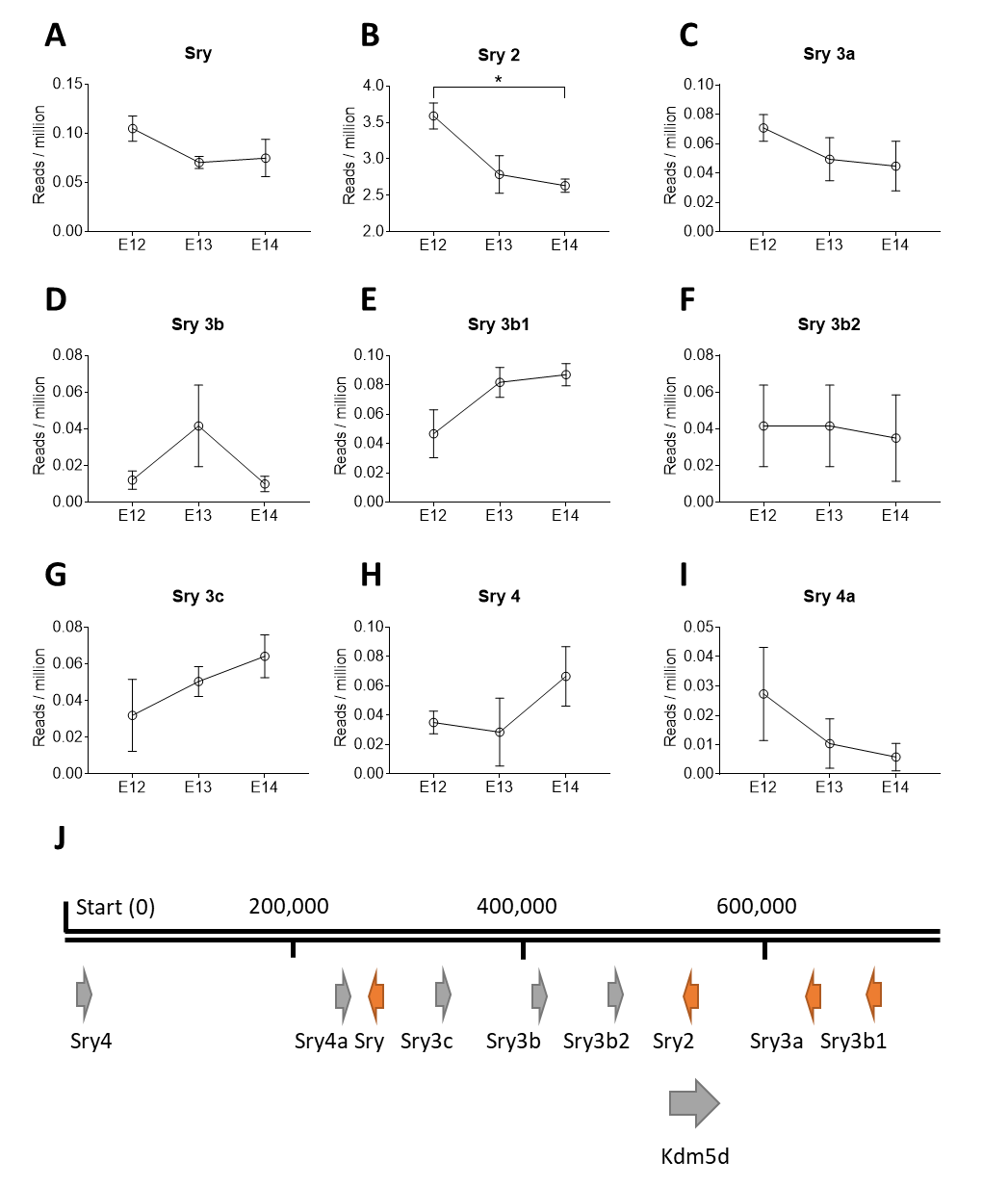


**Supplementary Figure S2.** Copy number and localization of *Sry* genes on the Y chromosome. (A-I) The reads per million of *Sry* homologues, are shown. Student’s t-test was utilized to determine significant differences between stages. One way ANOVA followed by Tukey’s multiple comparison post-test for expression data using the GraphPad Prism 8 software. (n=3, *p<0.05). (J) The location and orientation of *SRY* homologues and the Kdm5d gene on the Y chromosome are summarized.


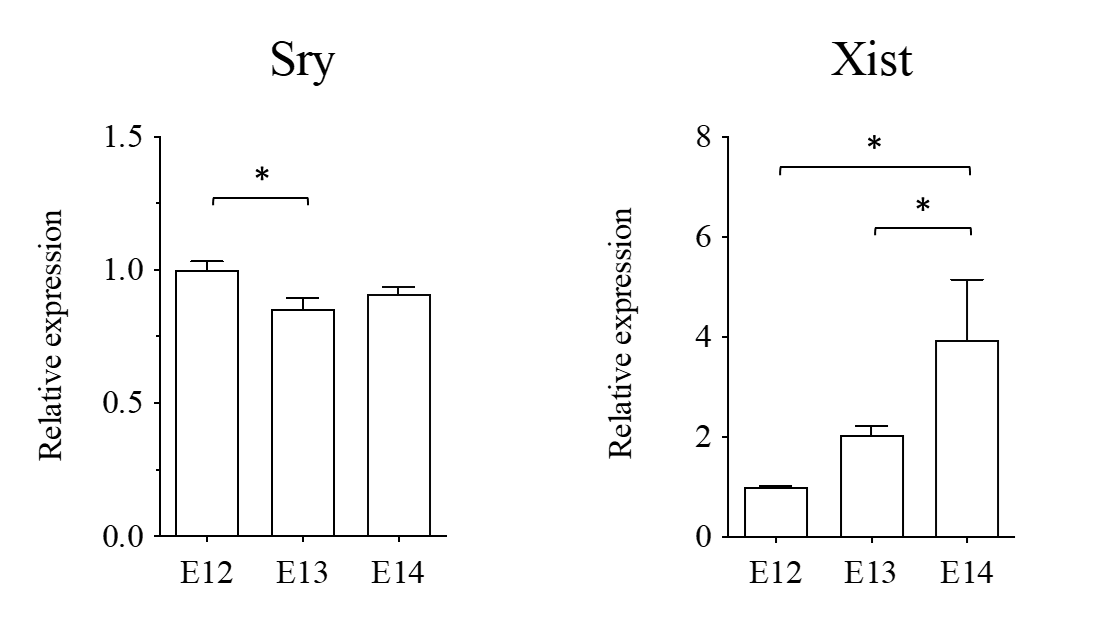


**Supplementary Figure S3**. qPCR validation of brain specific expression of *Sry* and *Xist* genes. Statistical analysis was performed using One way ANOVA followed by Tukey’s multiple comparison post-test for expression data using the GraphPad Prism 8 software. (n=3, *p<0.05).


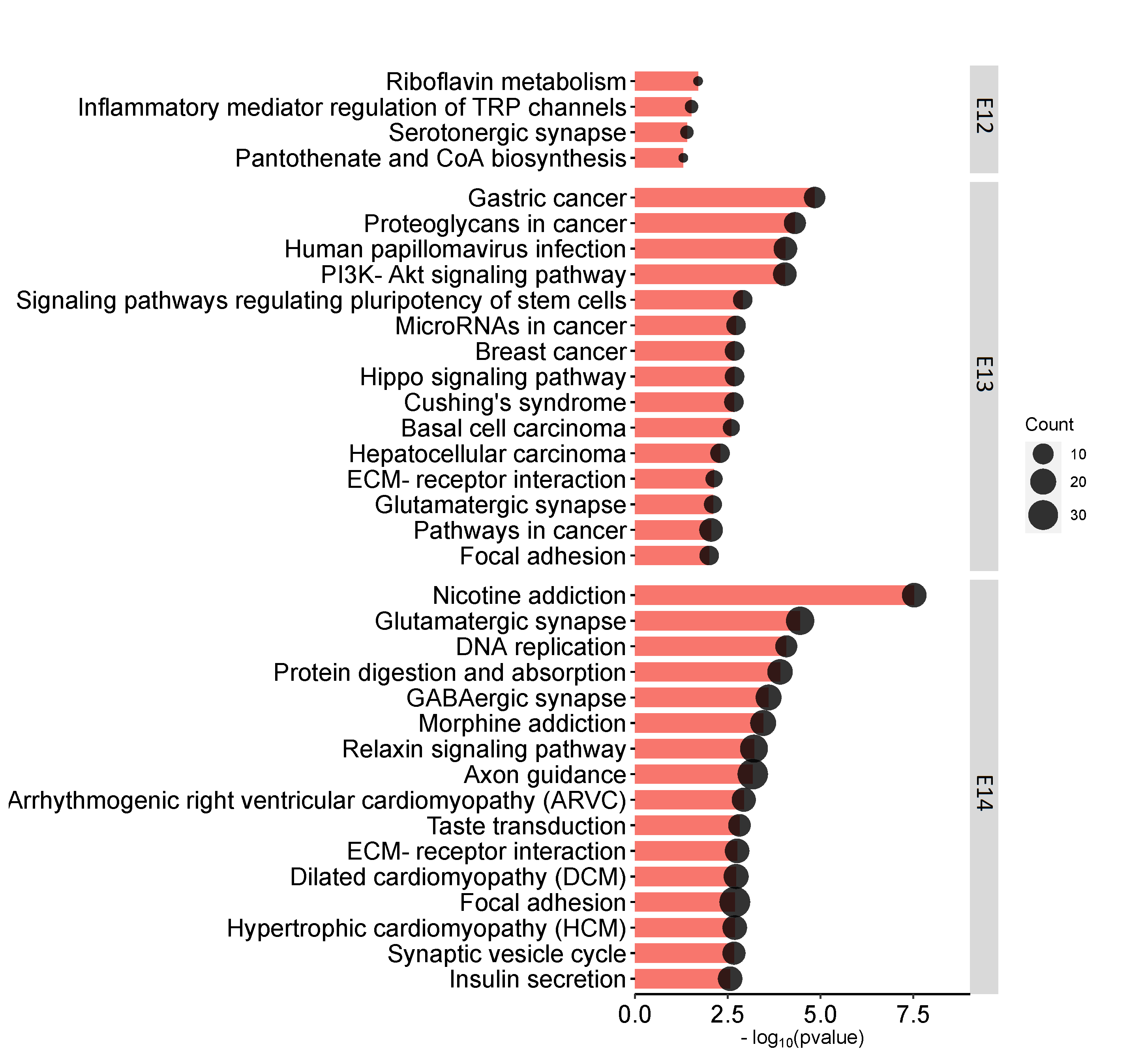


**Supplementary Figure S4.** Enrichment of KEGG pathways among DEGs from E12 to E14. The enriched pathways are ranked based on their p-values, providing a measure of statistical significance. The size of the dots in the plot corresponds to the number of genes associated with each enriched pathway, offering a visual representation of the extent of gene involvement in each pathway.


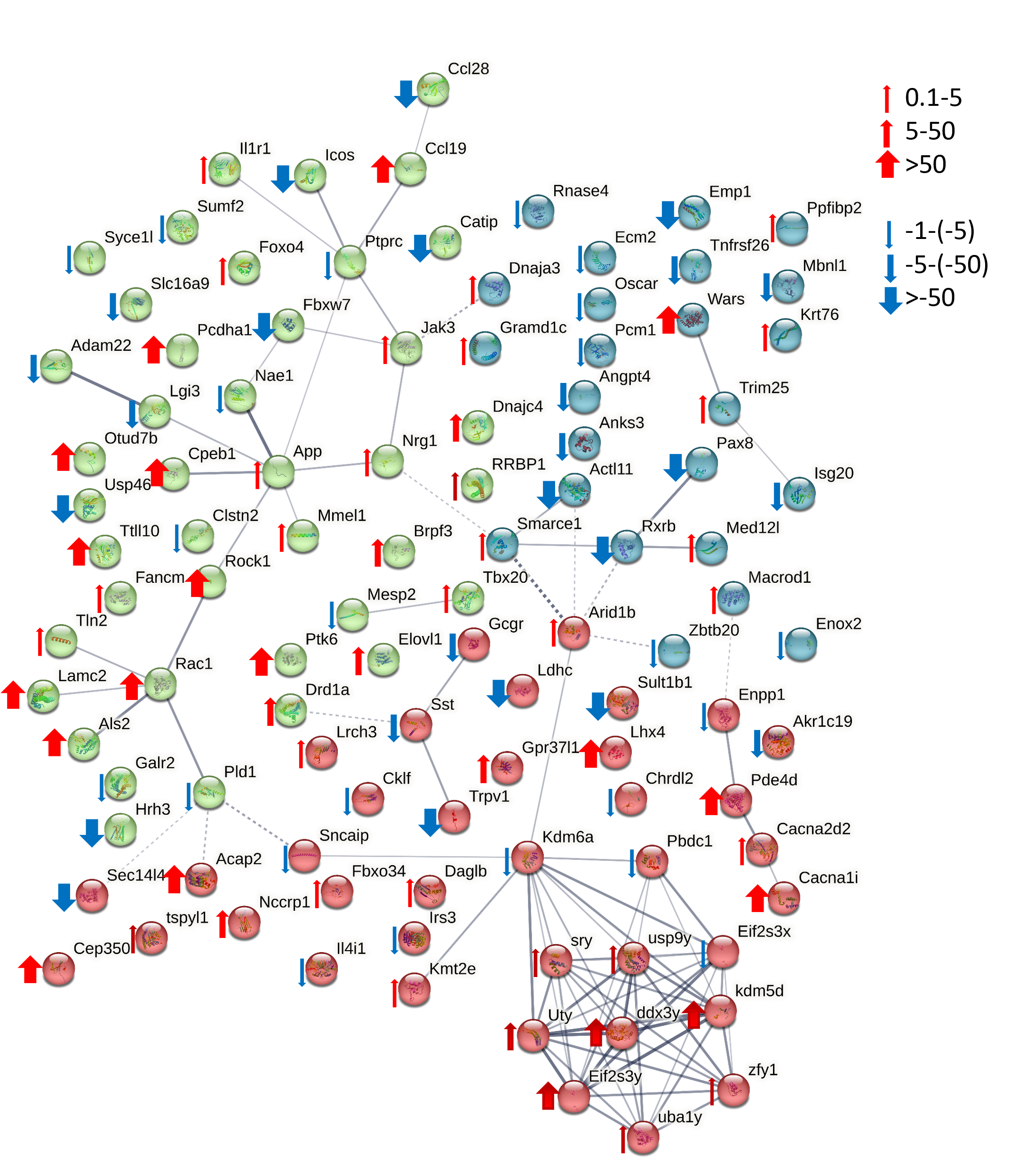


**A**


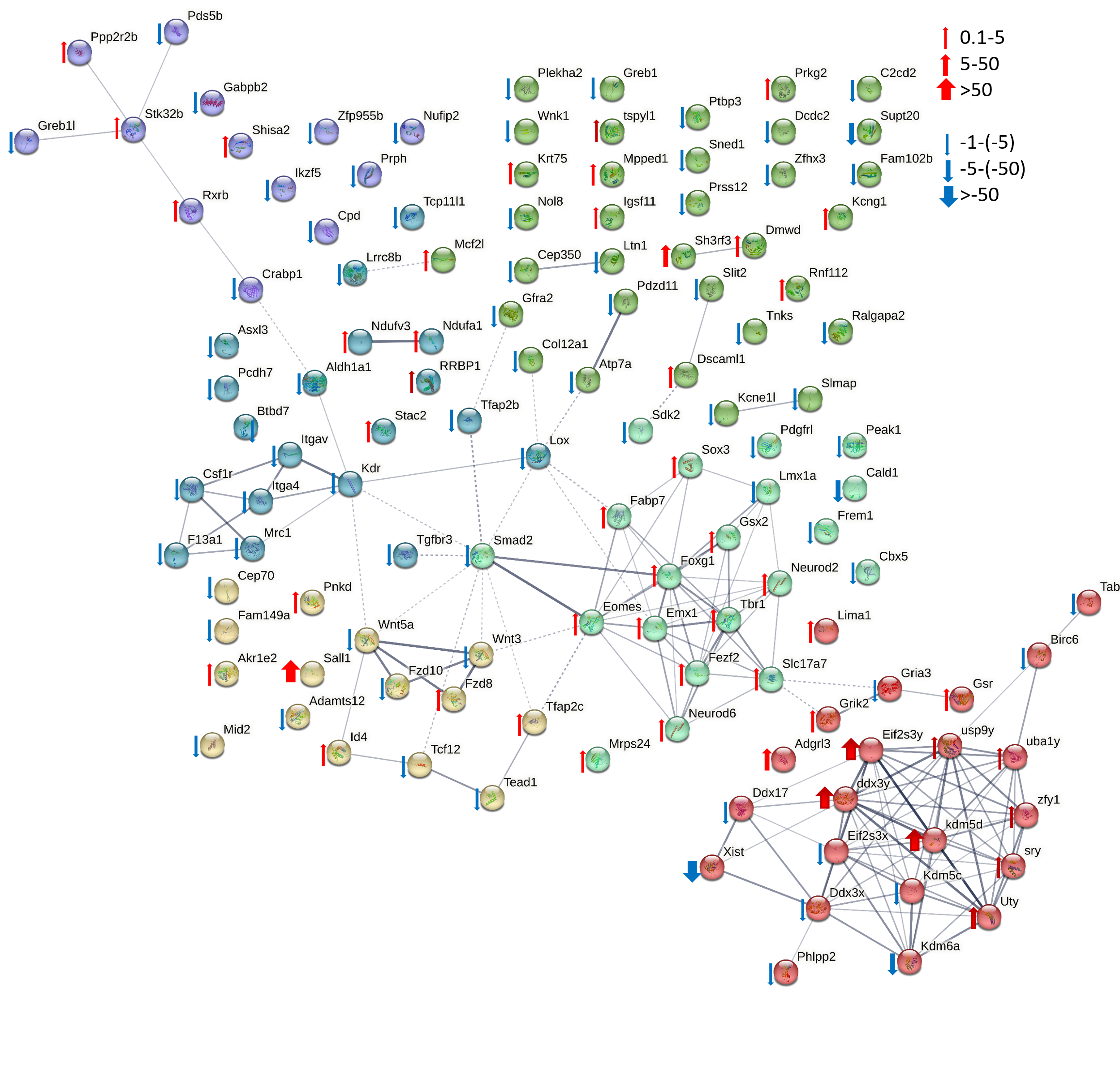


**B**


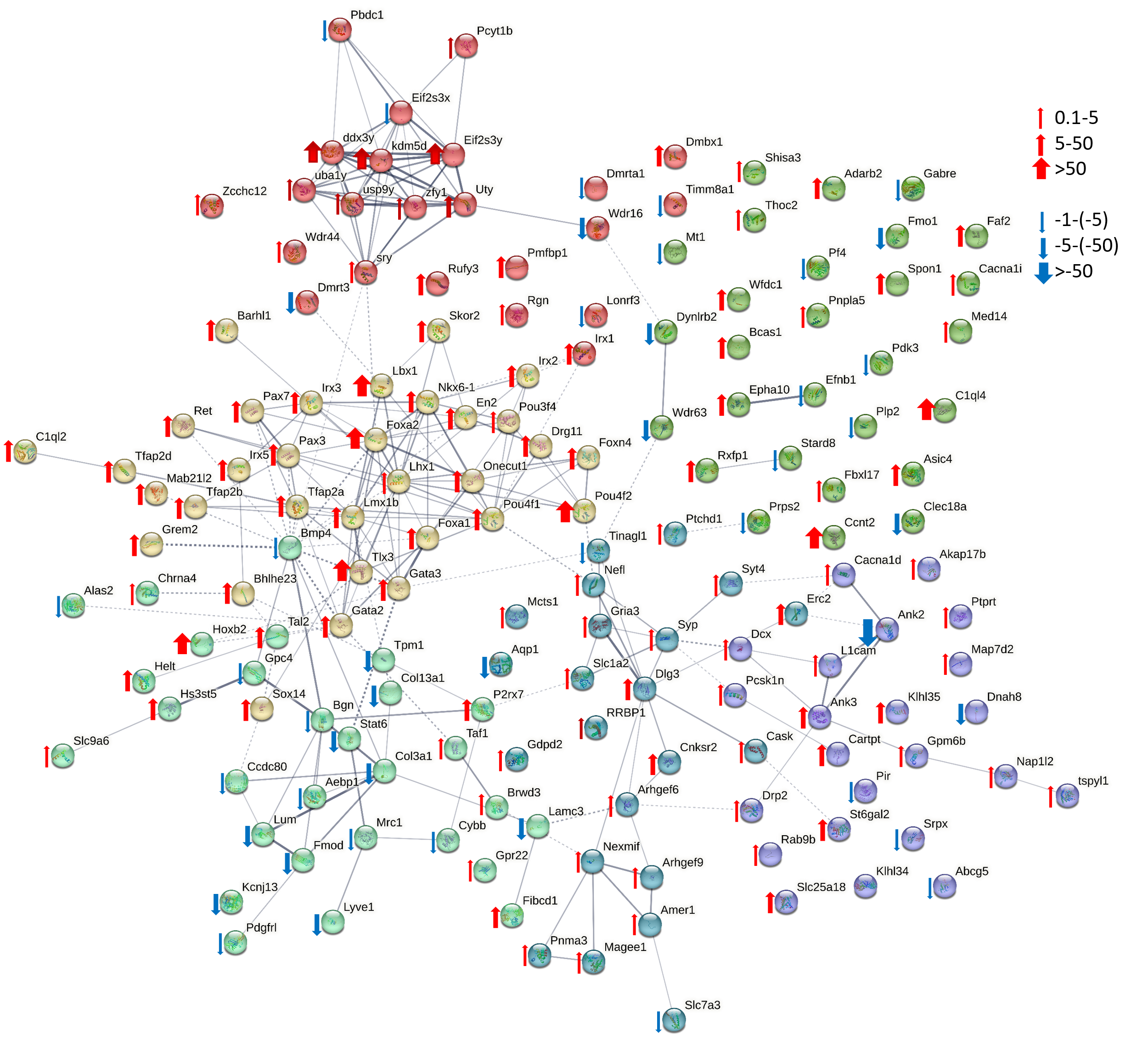


**C**

**Supplementary Figure S5.** Functional gene network analysis of neural development genes at (**A)** E12, (**B)** E13, and (**C)** E14 with STRING. Each node represents a gene, and a connecting line (edge) represents the interactions among the genes. (connecting lines). The thickness of the edge represented the strength of interaction. Nodes are color coded as black, blue and white based on their functional clustering (k-means). Differential regulation of the genes was shown by red (upregulated), and green (downregulated) halo and the thickness represent the fold change. Gene count was shown for Y-linked genes. All networks had a significant enrichment p-value less than 1e-16 and comprised of 102, 123, and 157 nodes, and 62, 168, and 236 edges, for E12, E13, and E14, respectively. Nodes had an average degree of 1.22, 2.73, and 3.01 and clustering coefficient of 0.30, 0.39, and 0.34 for the respective stages.


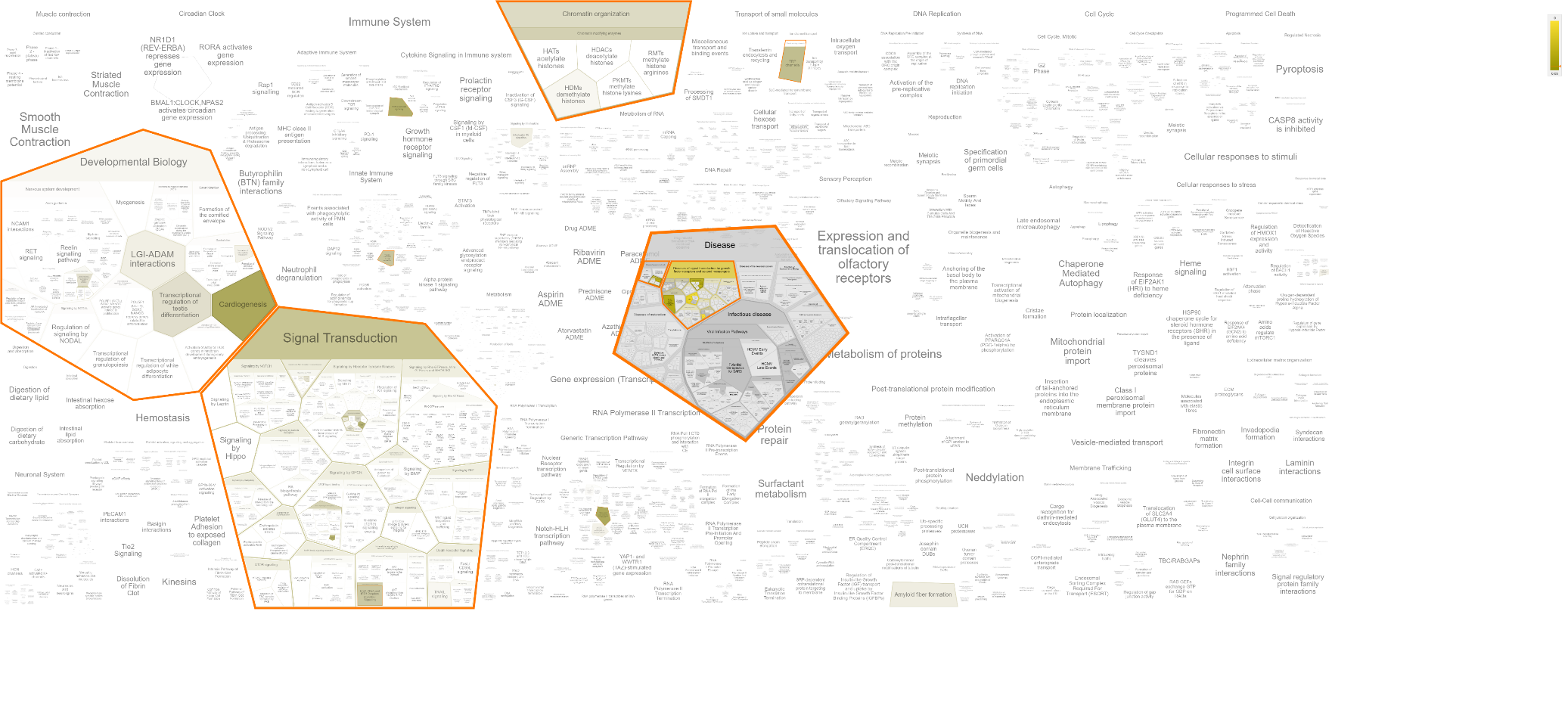


**A**


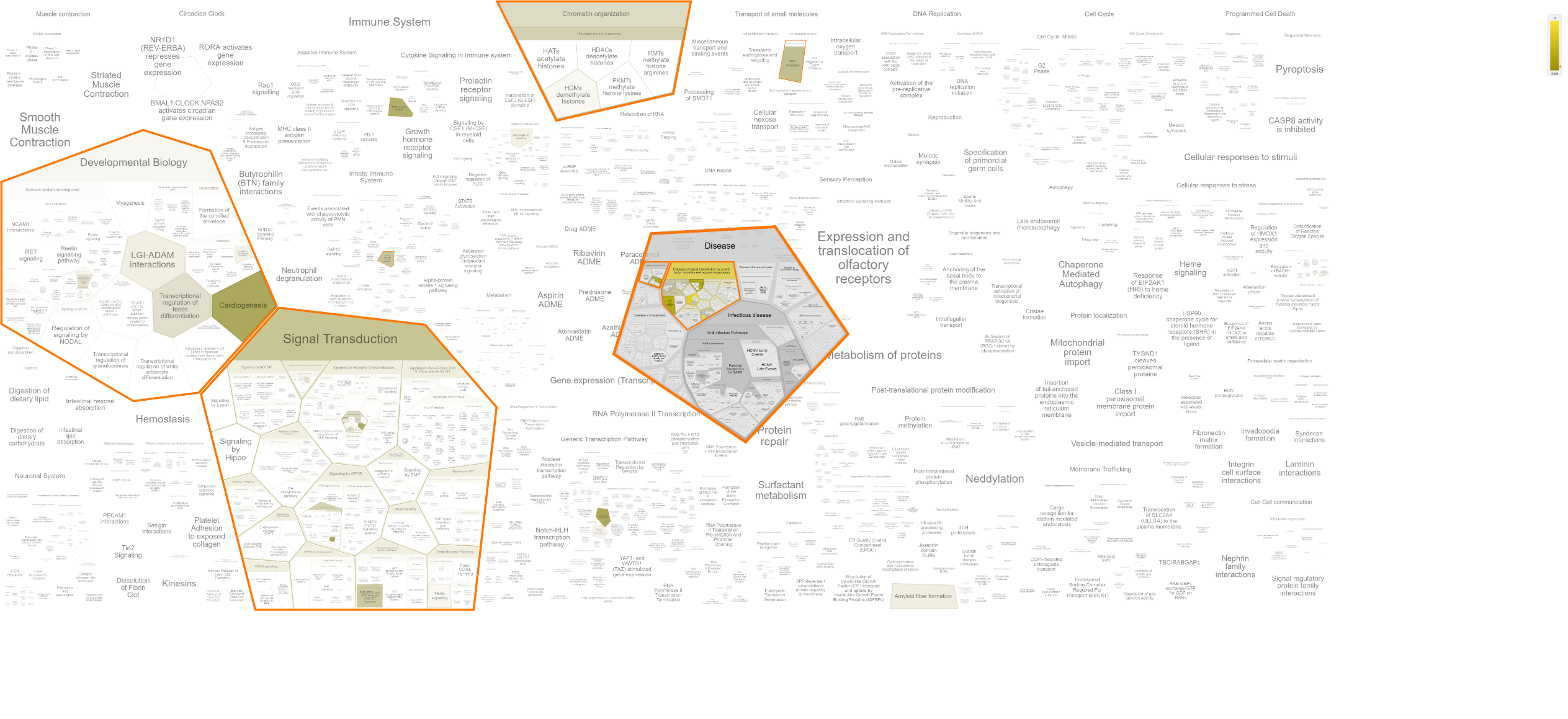


**B**


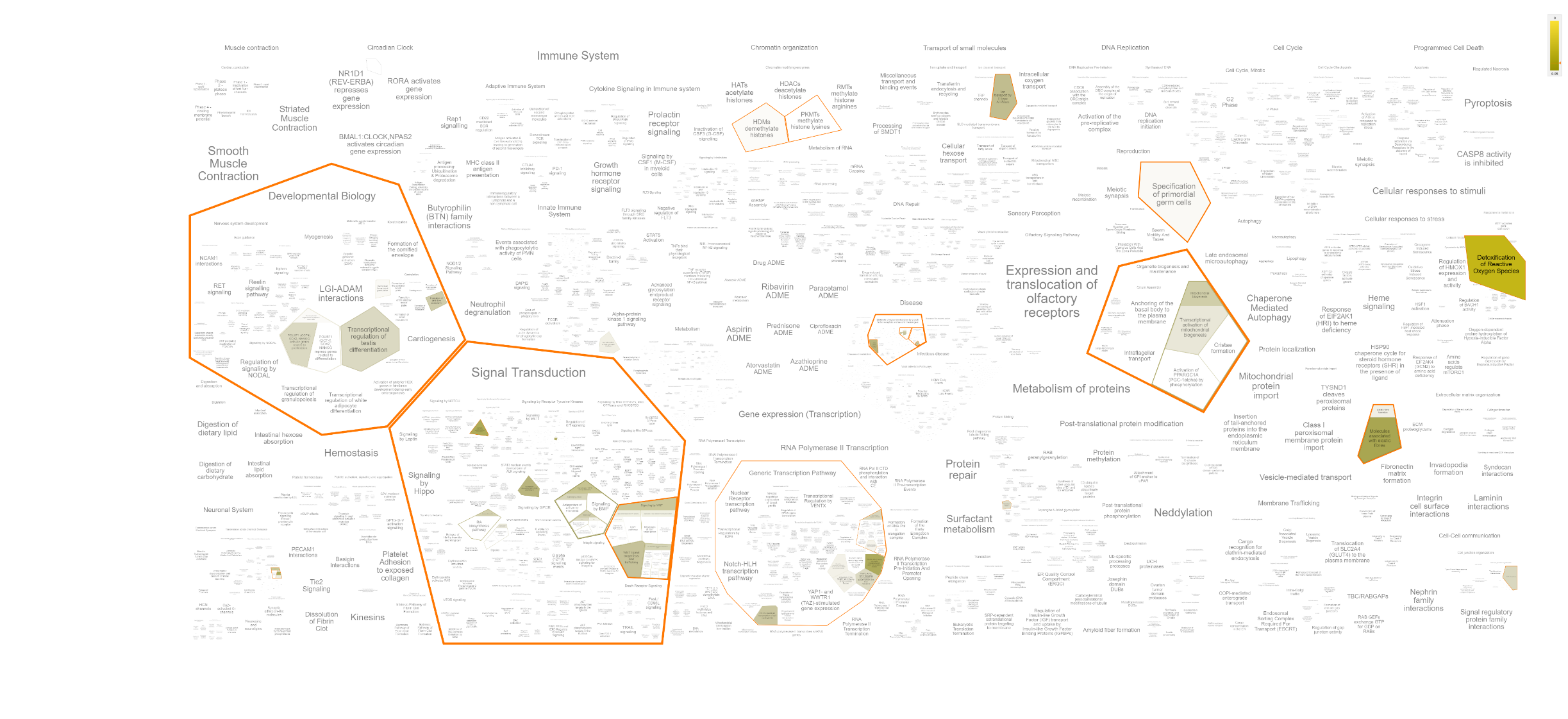


**C**


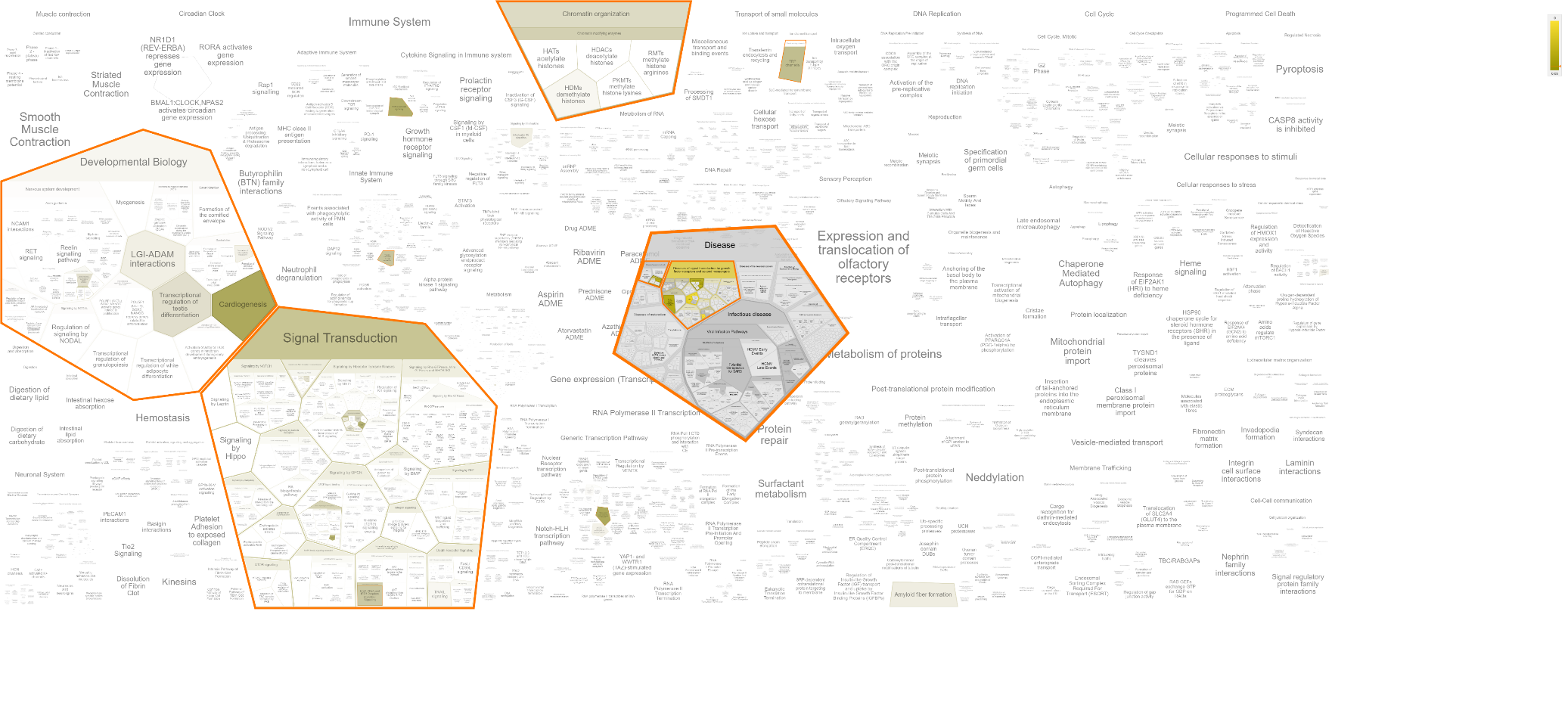

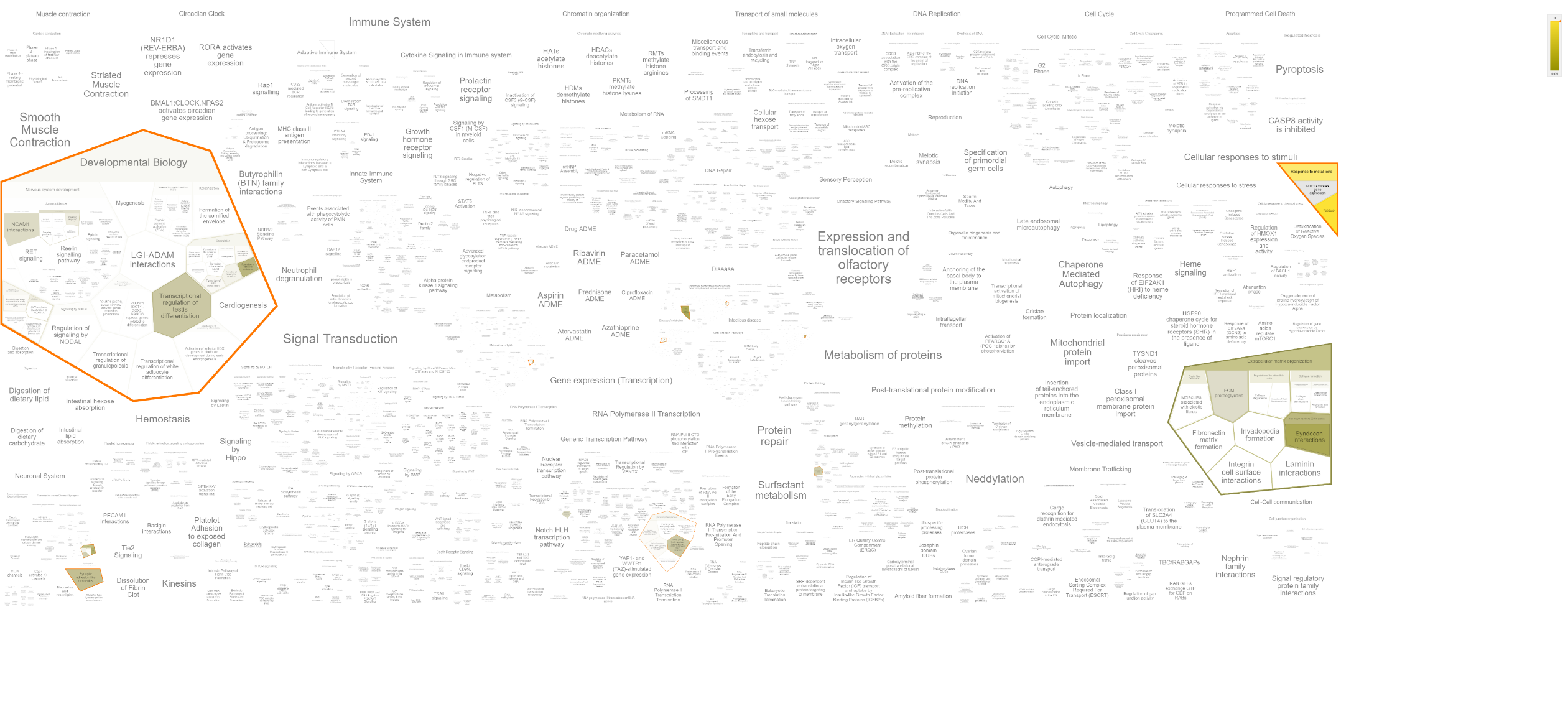


**Supplementary Figure S6.** Reactome Voronoi treemaps of the top enriched pathways. The figures show pathways influenced by genes showing high differential expression at (**A**) E12, (**B**) E13 and (**C**) E14 in male rat brain.
